# Climate change and the sustainable use of medicinal plants: a call for “new” research strategies

**DOI:** 10.3389/fphar.2024.1496792

**Published:** 2025-02-03

**Authors:** Olha Mykhailenko, Banaz Jalil, Lyndy J. McGaw, Javier Echeverría, Marce Takubessi, Michael Heinrich

**Affiliations:** ^1^ Pharmacognosy and Phytotherapy, UCL School of Pharmacy, London, United Kingdom; ^2^ Pharmaceutical Chemistry Department, National University of Pharmacy, Kharkiv, Ukraine; ^3^ Phytomedicine Programme, Department of Paraclinical Sciences, Faculty of Veterinary Science, University of Pretoria, Pretoria, Gauteng, South Africa; ^4^ Departamento de Ciencias del Ambiente, Facultad de Química y Biología, Universidad de Santiago de Chile, Santiago, Chile; ^5^ Pharmacy Department, Health Polytechnic of the Ministry of Health Kupang, Kupang, Indonesia; ^6^ Department of Pharmaceutical Sciences and Chinese Medicine Resources, Chinese Medicine Research Center, College of Chinese Medicine, China Medical University, Taichung, Taiwan

**Keywords:** endangered medicinal plants, ecosystem factors, climate change, sustainable practices, conservation strategies, traditional medicine, ethnopharmacology, key sustainability indicators

## Abstract

Climate change and human activities severely impact the viability of plants and ecosystems, threatening the environment, biodiversity, and the sustainable development of plant-based products. Biotic and abiotic (ecosystem) determinants affect species distribution and long-term survival, which in turn influence the quality of plants used as herbal medicines and other high-value products. In recent decades, diverse anthropogenic impacts have significantly affected these quality aspects. Climate change, excessive plant exploitation, habitat loss, species vulnerability, and other factors have adversely affected the growth, reproduction, and adaptation of species populations, as well as the quality and volume of primary plant materials supplied to pharmaceutical markets. Despite these growing challenges, there is limited knowledge of potential strategies to prevent or mitigate these impacts, particularly for vulnerable species collected from the wild or harvested from traditional production systems. Hence, effective strategies for preserving and increasing plant populations are urgently needed. In this study, we propose a new framework including the main sustainability factors to better understand and address the vulnerability of a species, hence mitigate the impact of climate change. We assess the applicability of our proposed framework via seven case studies of vulnerable species (i.e., *Aquilaria malaccensis* Lam., *Boswellia sacra* Flück., *Crocus sativus* L., *Panax quinquefolius* L., *Pilocarpus microphyllus* Stapf ex Wardlew., *Rhodiola rosea* L., and *Warburgia salutaris* (G.Bertol.) Chiov.) from main biogeographic realms, all widely used as medicinal plants. These species present various challenges related to the sustainability of their use, impacting their current and future status locally and globally. Their economic importance, combined with rising demands and specific risks of overexploitation, are also key factors considered here. The suggested framework for the sustainability of medicinal and other high-value plant-based products in the phytopharmaceutical industry emphasises strategies that promote conservation and sustainable resource use. It can also be adapted for other vulnerable species requiring urgent attention.

## 1 Introduction

Plants are a rich source of unique primary and secondary metabolites, many of which serve as medicines. They play a critical role as key ingredients in pharmaceuticals, functional/health foods, cosmetics, fragrances, agrochemicals, flavours, colouring agents, spices, biopesticides, and general food additives. There has been a dramatic growth (Howes et al., 2020) in demand for such ingredients not only in established economies but also in fast-emerging markets, for example, the People’s Republic of China, Middle Eastern countries, India, Brazil, México, and South Africa (Rezaie et al., 2012; Carvalho et al., 2018; Qu et al., 2014; Liu et al., 2023). Despite the strict quality control standards for medicinal plants in pharmacopoeias (Heinrich, 2015), there is no systematic framework for ensuring sustainable practices in sourcing current and future medicinal and other high-value plant-based products, nor to address the critical research and development needs in the context of climate change.

Dramatic changes to the global climate (Young et al., 2015), which have accelerated enormously over the last few decades, strongly impact the supply and sourcing of wild plant populations and cultivated resources, especially the established and traditional production systems (Applequist et al., 2020; Schindler and Hilborn, 2015; Chen et al., 2016). The dangerously unsustainable rates of anthropogenic damage–to the atmosphere, topsoil, forests, freshwater, ocean resources, and biodiversity–were central concerns of the first “*World Scientists’ Warning to Humanity*” (1992) (Ripple et al., 2017). Despite this, there remains a lack of broad societal understanding of our responsibilities and insufficient scientific methods and tools to evaluate how the production and trade of medicinal/health food plants (MHFPs) contribute to climate change and how climate change impacts their sustainable sourcing. This includes effects on supply, costs, quality, and consumer acceptance of MHFPs. This disruption may lead to reduced production of herbal medicines or increased risk of adulteration. Furthermore, the impacts of climate on plant resources and their implications for pharmaceutical supply chains have not been systematically documented. The medicinal plant sector needs to assess its environmental footprint, especially regarding how production and sourcing may contribute to or mitigate climate change (Howes et al., 2020; Pacifici et al., 2015). Unlike other areas, such as animal protection, where research and actions have been prioritised (Advani, 2023; Patni et al., 2022), the environmental impacts of MHFP sourcing and production require a more dedicated focus.

Previous studies, including surveys, frameworks, and systematic analyses of published data, have mainly focused on the problem of climate change and plants (Young et al., 2015; Eckardt et al., 2023; Parmesan and Hanley, 2015; Abbass et al., 2022; Ali et al., 2024), tracking population changes among populations of vulnerable species in this context (Pacifici et al., 2015; Pearce-Higgins et al., 2022; Yesuf et al., 2021) and exploring regionally restricted conservation strategy (Chen et al., 2016; Feigin et al., 2023; Wang et al., 2024a; McLaughlin et al., 2022) to save plants and biodiversity (Howes et al., 2020; Advani, 2023; Wang et al., 2024a; Mori et al., 2024). However, no comprehensive approach enables researchers to determine the critical stages of a species’ status or vulnerability in the context of pharmaceutical/health food needs, including the harvesting of high-quality and pharmacognostically well-defined primary material. The framework for developing the necessary advancements based on evidence-based scientific approaches to achieve sustainability and the conservation of specific species is poorly developed. Contrary to high-value, large volume food crops, medicinal plants are typically high-value, low-volume products (Atanasov et al., 2015; Booker et al., 2012). Sustainably managing MHFP is essential for both biodiversity conservation and ecosystem resilience. Ecosystem services (Reid et al., 2005; Hawken et al., 2021), including climate regulation and pollination are strengthened by diverse plant ecosystems.

The concept of ecosystem services focuses on the benefits derived from nature, allowing an evaluation of different benefits beyond strictly economic or environmental trade-offs alone. This approach requires a comprehensive understanding of ecological functions, sustainable practices, and socio-economic dynamics. Adapting research strategies to focus on ecosystem services is essential, as this approach integrates ecological, economic, and social factors, supporting sustainable development and the long-term availability of plant-based resources. By aligning research strategies with sustainability goals, we can help protect biodiversity, support human health, and strengthen resilience in the face of climate change. Here, we focus on utilitarian aspects, but ecosystem services also include the cultural importance of these species and the environments from which they are extracted (Milcu et al., 2013; Daniel et al., 2012).

## 2 The proposed framework

**FIGURE 1:**
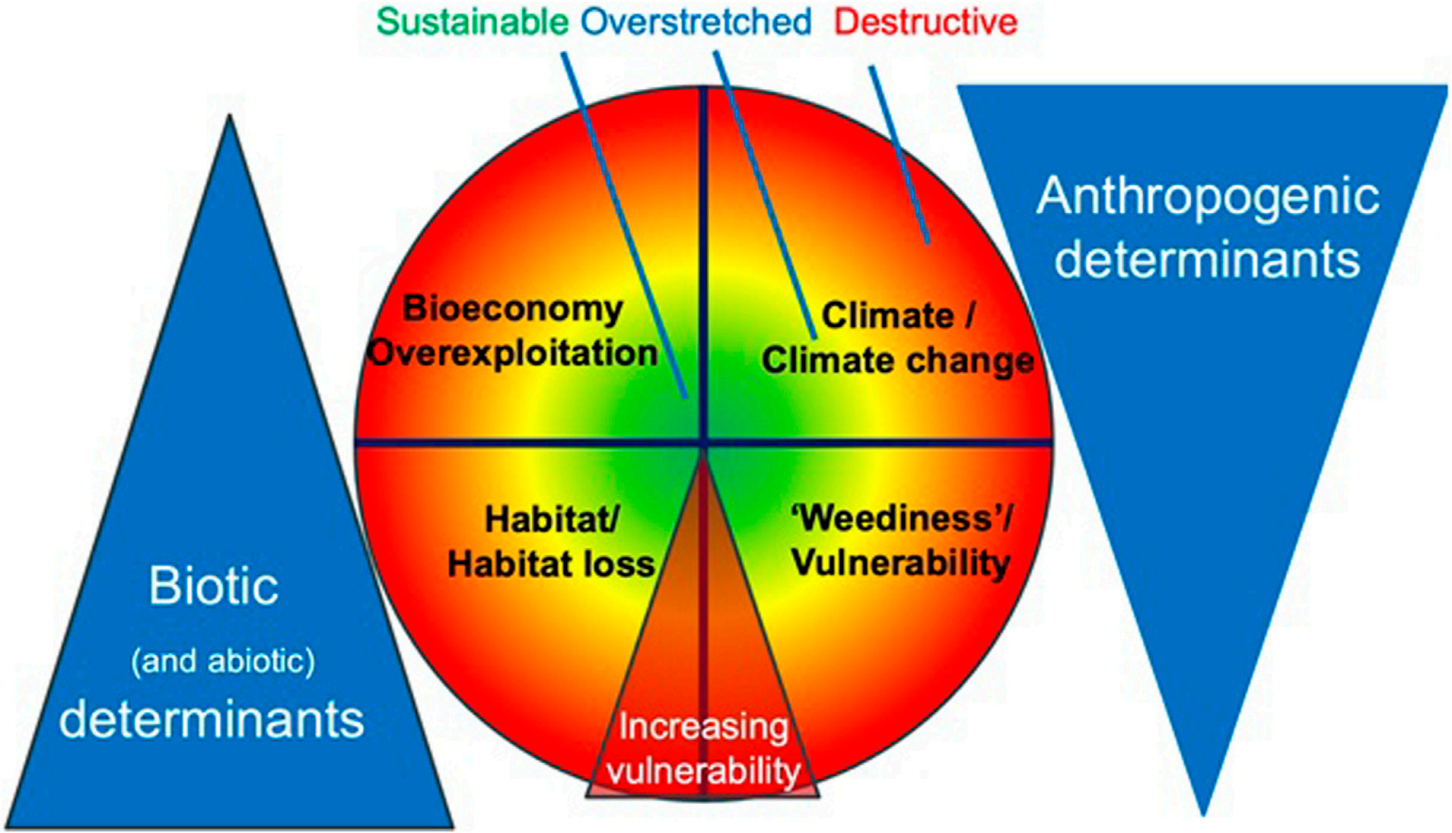
The conceptual framework for addressing the degree of a vulnerability of a species based on selected sustainability determinants in the context of climate change, incorporating a spectrum from biotic to anthropogenic determinants.

In this study, we propose a framework to redefine research priorities in the research and development of MHFP. Our proposed conceptual approach addresses the research and development needs linking specific actions for specific species and suggests main environmental factors that influence species abundance, spanning biotic and anthropogenic determinants (Figure 1). For instance, increased species vulnerability–represented by colour changes in the central circle–arises under the influence of biological and anthropogenic determinants. These broader determinants can be understood based on significant shifts in one or more of the four specific determinants of a species’ sustainability: “habitat/habitat loss”; “reproductive success”; “climate change”; and “bio-economy (uses)”, which we propose as key sustainability indicators for addressing a species vulnerability in the context of climate change (Figure 1). We define three levels of concern (with a simple colour coding) to assess the degree of a species’ vulnerability (Table 1), which, of course, represents a continuum of risks.

**TABLE 1:** Degree of vulnerability of a species (**key sustainability indicators**) in the context of climate change defined using three levels of concern (colour coding). All levels are estimates based on complex datasets and they provide a qualitative orientation.

Green	Sustainable—indicates a balanced development of the species, with favourable current characteristics and reliable positive trends for future generations
Yellow	Overstretched—indicates that the species is at risk of deteriorating due to the influence of one or more environmental factors
Red	Destructive—risk of an extreme degree of vulnerability of the species

While the proposed framework will not directly influence climate change or policy in the short term, it provides the necessary scientific foundation and strategic direction to drive future advancements in these areas. By outlining core research priorities, the framework aims to support impactful changes that align with long-term goals for environmental sustainability, research and policy development Centered around the Sustainable Development Goals (SDGs). The focus here is on species that are of major economic importance in the respective regions and which are core elements within the regional environments (i.e., they can be conserved using locally suitable strategies). While the focus here is on the vulnerability of individual species, any change to a species abundance clearly impacts both the wider ecosystem in a region and the communities using these resources (both positively and negatively).

While risks are often associated with the overexploitation of resources such as specific species or a habitat, developing a research strategy requires a clear distinction between biological (natural) and anthropogenic determinants. These biological determinants include biotic and abiotic elements related to a species’ habitat, such as the extent of its distribution and its reproductive capacity. Key considerations involve the physical environment and the specific biological conditions for a species’ survival and successful reproduction. Abiotic determinants are not considered here in detail, since, while important, they can generally not be influenced. Anthropogenic determinants include changes in production systems related to climate, climate change, and the demand for specific species as ingredients. These anthropogenic (or human) determinants are closely interconnected with biotic (and abiotic) determinants that modulate the ecosystem, each having the potential to impact the other (Figure 1). Understanding these interactions is crucial for creating a comprehensive research strategy that safeguards ecological balance and promotes the sustainable use of plant resources.

The first key biotic (and abiotic) determinant is habitat, encompassing the natural environment where species usually live. In this context. It also includes environments created through human activities like agricultural production systems and secondary forests. Understanding both the global abundance of materia prima and the impact of human-induced habitat changes is essential for assessing climate change effects on resource utilisation. Habitat shifts–driven by urbanisation, agriculture, or climate change–can directly influence the availability and sustainability of plant-based resources. The second core biotic determinant is a species’ vulnerability, which is closely tied to reproductive ability (weediness) and adaptability to changing conditions as well as to its habitat. Medicinal plant species such as *Hypericum perforatum* L. (St. John’s wort) demonstrate high reproductive success (Koperdáková et al., 2004; Barcaccia et al., 2006), making reproductive ability a vital criterion for assessing ecological risks and resilience. While this reproductive strength can offer a buffer against environmental threats, it also underscores the need to balance the risks and benefits associated with its use and cultivation.

Two anthropogenic determinants are crucial: one is a species response to climate change. In other words, the impact of increasing average temperatures, changes to the rainfall pattern, and lower minimum temperatures. The second key biotic determinant, influenced by climate and impacting plants directly, is insect activity, which significantly affects plant populations. For example, orchid species such as *Cephalanthera rubra* (L.) Rich. and *Cephalanthera longifolia* (L.) Fritsch (Taura and Gudžinskas, 2024) fruited abundantly in the second half of the 20th century. However, their recent population decline is believed to be linked to climate change. One possible explanation is a desynchronisation between flowering times and pollinator activity (Pyke et al., 2016). Detailed studies of flowering phenology and pollinator dynamics across the species’ range are essential to validate this hypothesis.

Lastly, the bioeconomy, or the level of resource use (including overuse), is a crucial aspect to consider. This covers all elements related to production *per se* and how resources are utilised within both global and regional economic frameworks. Two major drivers stand out: first, the direct destruction of habitats due to urban expansion and other land-use changes, which reduce the availability of natural resources. Second, the recent surge in demand for certain “boom” products over the last 3 decades, fueled by internet-based commodification, such as roseroot (*Rhodiola rosea* L.), elder (*Sambucus nigra* L.), and maca (*Lepidium meyenii* Walp.). Understanding which production systems and value chains (Booker and Heinrich, 2016) can minimise the negative impact of a species’ use within the global bioeconomy is crucial. This includes addressing challenges associated with botanical drugs like root/rhizome and bark, which are particularly vulnerable to overharvesting, and commercialisation is vital for maintaining both ecological balance and economic viability.

**FIGURE 2:**
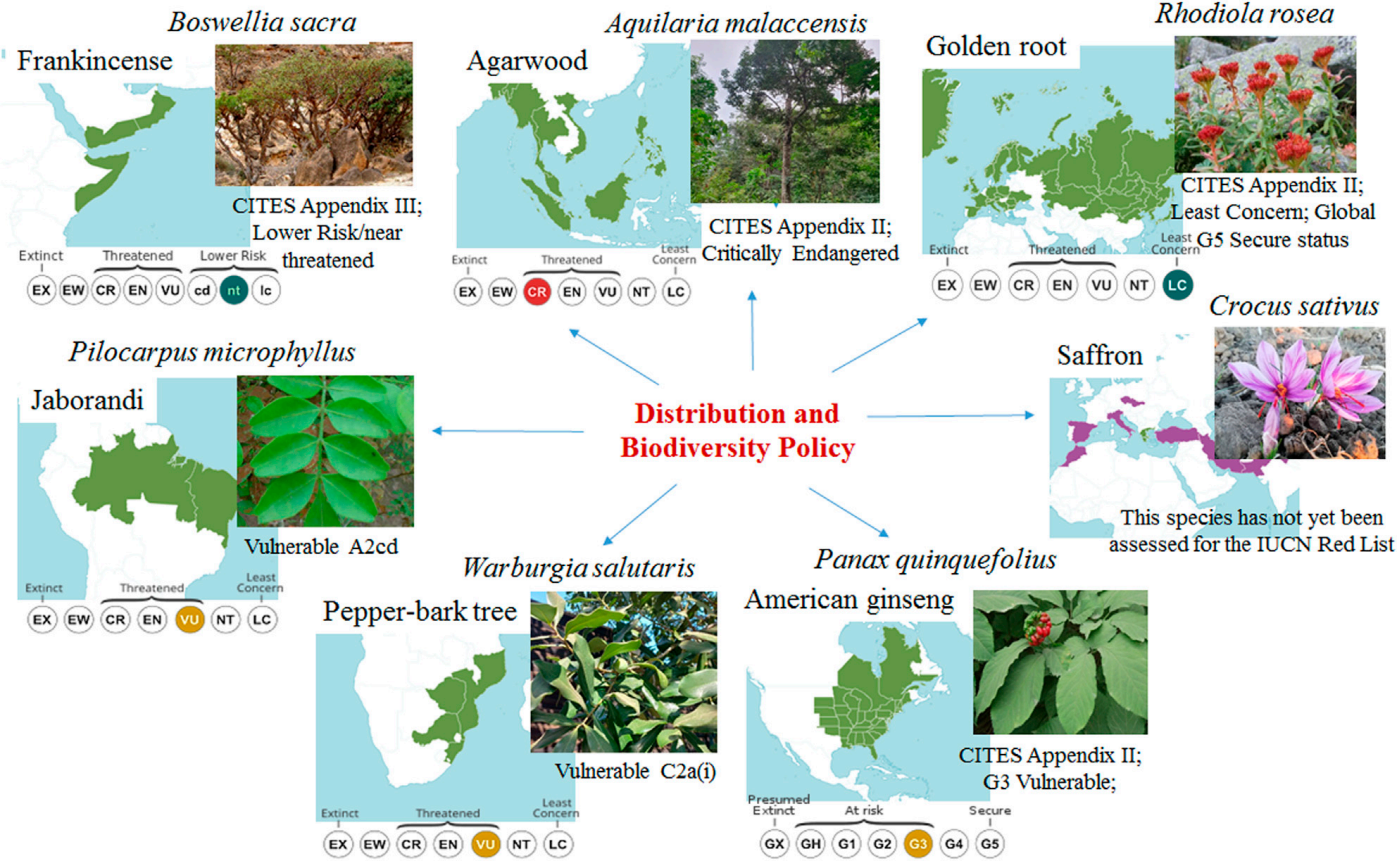
Overview of the seven selected case studies. The selection criteria for these species were: 1) The different challenges related to their use that impact on their current and future status at both local and global levels; 2) The different biogeographic realms; 3) The economic importance of the species, wide use, combined with increasing demands and particular risks of overexploitation (UN Environmental Programme, 2024; Convention on International Trade in Endangered Species CITES, 2024; CITES, 2024; IUCN, 2021).

To evaluate the applicability of the proposed framework, we selected seven case studies of vulnerable species: *Aquilaria malaccensis* Lam., *Boswellia sacra* Flück., *Crocus sativus* L., *Panax quinquefolius* L., *Pilocarpus microphyllus* Stapf ex Wardlew., *Rhodiola rosea* L. and *Warburgia salutaris* (G.Bertol.) Chiov.) (Figure 2). These species present unique, and example challenges related to their use, affecting their current and future status at both local and global scales. The main selection criteria included here are their presence across different biogeographic realms, economic importance, widespread use, rising demand, and specific risks of overexploitation (Figure 2).

The seven case studies are categorised using the three levels of concerns shown in Table 1, with individual Tables provided for each case study in their respective sections. Through this framework, we evaluate the available knowledge on each species in relation to the four main sustainability determinants in the context of climate change and their implications for redefining research and development strategies (Figure 2; Tables 2–8).

**TABLE 2:** *Aquilaria malaccensis* Lam. as a case study for vulnerable plant species in SE Asia/Oceania; for the three levels of concern, see Table 1.

Species name		*Aquilaria malaccensis* Lam., Thymelaeaceae, agarwood	
Botanical drug used		Aromatic resin and wood	
Uses in medicine		Anti-inflammatory (Alamil et al., 2022). Gastric ulcers, for the treatment of abdominal pain and as a sedative (Wang et al., 2021)	
Conservation status		CITES Appendix II (IUCN, 2024)	
		Critically Endangered (IUCN 3.1)	
Habitat/habitat loss	Reproductive success	Climate change	Bio-economy (uses)
*A. malaccensis* is a tropical tree that grows in high-rainfall areas throughout humid regions (Thompson et al., 2022). The species depends on consistent rainfall for optimal growth	The species is characterised by low regeneration. A long-term, extensive demographic study shows that a primary forest’s seedling stocking by natural regeneration is inadequate. Moreover, with a reproductive size requirement of approximately 10 cm dbh, tree densities >10 cm dbh are marginally less than one tree per hectare, and the juvenile-to-adult ratio is barely 1.5 (Ministry of Environment and Forestry B, 2024). Over 6 years of observation, the wood diameter increased by only 1 cm per year on average. The natural regeneration rate under mature trees in the primary forest is low, being only 3–5 juveniles per tree (Ministry of Environment and Forestry B, 2024)	Altered temperatures and precipitation can lead to traditional growing areas of *A. malaccensis* becoming unsuitable, leading to habitat loss. Take India as an example where climate change threatens the habitat suitability of Agarwood, with a decrease in suitable habitat of 34.28% under RCP4.5% and 14.64% under RCP8.5 by 2050, with a further decrease by 2070 (Sutomo and Kurniawati, 2021)	Agarwood is traded in various products and derivatives, including oil, wood, wood chips, flakes, powder, and carvings, where the quality of the resin determines the price and quality of these products. The major exporters of agarwood are Indonesia, Malaysia, and Thailand, with almost exclusively (more than 98%) wild stock exported from Malaysia and Indonesia. Wild populations of various *Aquilaria* species have declined considerably over the past 20–30 years (Soehartono and Newton, 2001)
		A geographical shift in Indonesia has been predicted with changes to suitable growth compared to current conditions (Borogayary et al., 2018). Temperature and rainfall are important factors in determining the vegetative and reproductive phenology of *A. malaccensis* (Borogayary et al., 2018). As a result, any climate change impacts on these variables may have a significant impact on its phenophases	Cultivation systems were developed to meet high demand, and their production has dominated the market compared to wild plants in 2017, 2019, and 2020

Assessment: The decrease in the wild population is mostly caused by over-exploitation, and cultivation with artificial introduction is an alternative way to meet the market demand for agarwood. Even though it has low regeneration, and is affected by climate change, the high demand in different bioeconomies drives the risks in terms of decreasing populations. Several studies show that if temperature and rainfall are modified in the future, natural habitats will be changed and potentially lost.

**TABLE 3:** *Boswellia sacra* Flück as a case study for vulnerable species in the Middle East; for the three levels of concern, see Table 1.

Species name	*Boswellia sacra* Flück, Burseraceae, frankincense, olibanum		
Botanical drug used	Resinous dried sap/resin		
Use in medicine	Immunomodulatory, neuroprotective, analgesic, antimicrobial, and anti-inflammatory		
Conservation status	CITES Appendix III, 2023		
	The IUCN Red List of Threatened Species: Lower Risk/near threatened, 1998		
Habitat/habitat loss	Reproductive success	Climate change	Bio-economy (uses)
The species is restricted mainly to Somalia, including outlying islands like Socotra (CITES, 1973; Thulin et al., 2019). The limited distribution poses a critical risk in terms of the species’ sustainable use (Johnson et al., 2022)	Slow growth rate and specific habitat requirements (rocky, well-drained soils) result in overall low reproductive success (Khan et al., 2018). Susceptibility to overharvesting can damage the tree, reducing its lifespan	The natural habitat in arid and semi-arid regions, primarily in Oman, Yemen, and Somalia, experiences increased temperatures and less precipitation patterns due to climate change. This impacts the growth and regeneration of *B. sacra* trees, as they are adapted to particular climatic conditions (Hamdiah et al., 2022). Rising temperatures and changing rainfall patterns may on inhibits affect its growth and resin production (Mwijuke, 2024)	The demand for frankincense has risen considerably [for *Boswellia* spp. in 1987 was 200–800 tonnes (DeCarlo et al., 2020) as compared to 2016-2017 was 1400–2000 tonnes]. *Boswellia* spp. grow in arid regions of Oman, Yemen, and Somalia (DeCarlo et al., 2020). These areas are characterised by harsh climatic conditions, including high temperatures and low rainfall (Amri and Shanfari, 2024). Habitat loss due to human activities such as land conversion for agriculture, urbanisation, and liverstock grasing is a significant threat to *B. sacra* (Eshete et al., 2012; Khan et al., 2022)
	Low seed germination under natural conditions (Hamdiah et al., 2022). *B. frereana* and *B. sacra* grow in arid regions of Oman, Yemen, and Somalia (Krystal, 2020). These areas are characterised by harsh climatic conditions, including high temperatures and low rainfall		

Assessment: *B. sacra* faces multiple threats from overexploitation, climate change, habitat loss, and biotic determinants. Sustainable harvesting practices, protecting its habitat, and involving local communities in conservation efforts are needed to ensure its survival.

**TABLE 4:** *Crocus sativus* L. as a case study for vulnerable species in Europe/Middle East; for the three levels of concern, see Table 1.

Species name		*Crocus sativus* L., Iridaceae. Saffron	
Botanical drug used		Stigma	
Uses in medicine		Antidepressant, anti-inflammatory, anti-atherosclerotic, antigenotoxic, and cytotoxic	
Conservation status		Not included on IUCN Red List	
Habitat/habitat loss	Reproductive success	Climate change	Bio-economy (uses)
*C. sativus* is a cultigen and as such the habitat is defined by the agricultural strategies to grow the species. It thrives in Mediterranean climates (Cardone et al., 2021) characterised by hot, dry summers and mild, wet winters. Changes to rainfall patterns and temperature cycles are key potential risk factors (Molina et al., 2005)	The low genetic diversity due to vegetative propagation increases susceptibility to diseases and environmental stresses (El Merzougui et al., 2024). The species is susceptible to corm rot (caused by fungi like *Fusarium* spp.), rodents, and insects (Zhang et al., 2022). Altered climatic conditions can render traditional saffron-growing areas unsuitable, leading to habitat loss	Increased temperatures, altered precipitation patterns, and extreme weather events can inhibits affect flowering and yield (Molina et al., 2005). Drought conditions, in particular, are becoming more frequent, natively affecting saffron’s growth cycle (Pirasteh-Anosheh et al., 2023). Long dry spells during the active growing months of the species lead to a decrease in the yield and quality of saffron (Ganaie and Singh, 2019). Significant decline in saffron production due to climate change, economic conditions, and farming practices, with some reports indicating a 60% reduction in crop area over 2 decades (Husaini, 2014)	There is a rise in demand, but core risks are based on climatic changes. Dominated by Iran, saffron production has seen significant changes from 2015 to 2023 (SunLand Saffron, 2024). Before 2023, in the Khorasan Razavi (Iran) the annual production of dry species was 350–370 tons, but the 2023 harvest was just 140 tons. Saffron productivity in India had declined from an average of 3.13 kg/ha to 2.61 kg/ha over the years before 2015 (Mir and Aasifa, 2023). In 2016-17, saffron production was about 2.80 kg/ha of dry spice per year, but in 2017–18 the average was just 0.973 yield/ha. Production plantation has declined from about 5707 ha to 3,715 ha in 2009–10 (Ganaie and Singh, 2019). In Castilla-La Mancha, Spain there is also a trend towards a decrease in dry raw material volumes from 7–9 kg/ha before 2022 and currently 3.5 kg/ha (Sam, 2024). In regions like Iran, Spain, and India, agricultural land is being converted for urban development, reducing the area available for saffron cultivation

Assessment: The dynamics of saffron production from 2015 to 2024 highlight the complex interplay of environmental, economic, and social factors impacting this high-value crop. While Iran managed to increase its production despite challenges, Kashmir saw a notable decline due to a combination of climate change, pollution, and economic difficulties. The most key and destructive factor affecting the sustainable cultivation of *C. sativus* is the climate, due to which there are shifts in the timing of planting/harvesting stigmas and, as a consequence, a decrease in the volume of saffron production.

**TABLE 5:** *Panax quinquefolius* L. as a case study of vulnerable species in North America; for the three levels of concern, see Table 1.

Species name	*Panax quinquefolius* L., Araliaceae, American ginseng		
Botanical drug used	Root		
Use in medicine	Adaptogen, uses as anti-inflammatory, antioxidant, antiviral, an alleged “anti-cancer” agent, among others		
Conservation status	G3 Vulnerable (NatureServe Explorer, 2023a); CITES Appendix II, from 1975 (Convention on International Trade in Endangered Species CITES, 2024)		
Habitat/habitat loss	Reproductive success	Climate change	Bio-economy (use)
*P. quinquefolius* is naturally, found in deciduous woodland in eastern and central Canada and the USA (McGraw et al., 2013; Government of Canada, 2015). While distributed over a wider area in woodlands, logging and land development (*cf.* bioeconomy) are primary drivers of habitat loss	Ginseng plants take several years to reach maturity, which slows recovery from overharvesting (Liu et al., 2021). Low seed production and high rates of seed predation further limit natural regeneration (Burkhart and Jacobson, 2004a). Ginseng is susceptible to diseases such as *Alternaria* leaf blight and root rot, which can devastate populations (Neils et al., 2021)	This species is sensitive to climate conditions, thriving in cool, temperate forests with well-drained soils. Optimal growing conditions include cool, moist, deciduous forests with temperatures of 10°C–15°C and high humidity (Schmidt et al., 2019; Van der Voort, 2005). Changes in temperature and precipitation patterns due to climate change will affect the distribution, reproduction, and health of ginseng populations. Warmer temperatures and changes in precipitation may lead to habitat loss and increased vulnerability to pests and diseases (Souther, 2011). Regional studies indicate that North-eastern U.S. forests may experience warmer temperatures and changes in precipitation, which could negatively impact on ginseng habitat (Kauffman, 2006a)	There is a high demand for wild ginseng, which is considered more medicinal than cultivated ginseng especially in China (USDA. China, 2022). The conversion of forest lands to agricultural lands disrupts their ecology and the habitat necessary for ginseng
			From January to November 2022, China imported 3 332.25 tons of American ginseng, a 90.56% increase compared to the same period in 2021, with an import value of USD 63.05 million, up 21.49% year-on-year (Baych, 2022). The majority of these imports come from Canada, which in 2021 accounted for 88.59% of the total import volume and 74.42% of the total import value (Baych, 2022). Poaching, driven by high market prices, exacerbates population declines and hinders conservation efforts. In particular, aaccording to report (Liu et al., 2021) the USA at 1970–1979 had harvested 12000,00 dry kg of ginseng while in 2010–2019 the procurement of plants amounted to only 254,100 dry kg

Assessment: American ginseng is a species of significant economic and cultural value, yet it faces numerous threats from overexploitation and habitat loss. The most vulnerable and dependent species are those affected by climate change. Sustainable management practices and conservation efforts are essential to ensure its survival for future generations.

**TABLE 6:** *Pilocarpus microphyllus* Stapf ex Wardlew. as a case study for vulnerable species in South America; for the three levels of concern, see Table 1.

Species name	*Pilocarpus microphyllus* Stapf ex Wardlew., Rutaceae, Jaborandi		
Botanical drug used	Leaves		
Uses in medicine	It is a source of pilocarpine used in glaucoma treatment		
Conservation status	Vulnerable A2cd		
Habitat/habitat loss	Reproductive success	Climate change	Bio-economy (uses)
*P. microphyllus* is widely distributed in the northern region of Brazil (Pinheiro, 1997) in open forest habitats (understory) in areas with higher light intensity, less dense forests, and frequently on rocky outcrops of Pre-Amazonian forest (Skorupa, 2000). The main threat to this species’ habitat is anthropogenic activity such as Amazon deforestation for agriculture and mining	The reduction in areas of suitable climatic conditions affects the food resources available to pollinators (Freimuth et al., 2022), threatening sexual reproduction and genetic variability of plants (Pinheiro, 1997). Chronic leaf harvesting potentially affects plant energy investment, survival, and long-term reproductive success. Techniques for cultivating this species remain incomplete and have not produced maximum productivity due to the few and fragmented studies conducted so far, focused mainly on pilocarpine variation (Abreu et al., 2007; Sawaya et al., 2011), *in vitro* cell culture (De Abreu et al., 2005), micropropagation (Sabá et al., 2002), propagation of seeds (Anaise Costa et al., 1970) and nutrients/pilocarpine relationships (Avancini et al., 2003)	Few studies have examined how climatic conditions can affect the growth, reproduction, and natural distribution (Monteiro et al., 2023). The expected climate changes (high atmospheric concentrations of CO_2_, high temperature, and water deficit) would negatively impact on the ecophysiology of the jaborandi, posing a threat of extinction for this species (Amaral et al., 2022)	Jaborandi’s productivity is around 1.8 t/ha/harvest. Considering between 5 and 8 annual harvests, this is between 9-15 t/year (Pinheiro, 1997). In 2010-2012, Brazil exported pilocarpine with an average trading volume of approximately 2,400 kg/year and an average yearly value of USD 6.4 million. During this period, the average price was USD 2600/kg (CNI, 2014)

Assessment: Jaborandi is important socioeconomically for many communities in northern and north-eastern Brazil. Public conservation and sustainable management policies must be undertaken soon to offset the effects of global climate change.

**TABLE 7:** *Rhodiola rosea* L. as a case study for vulnerable species in Asia, Europe and the entire holarctic; for the three levels of concern, see Table 1.

Species name	*Rhodiola rosea* L., Crassulaceae. Arctic root, golden root, rose root		
Botanical drug used	Root		
Use in medicine	Adaptogen, cancer prevention, anti-ageing, anti-inflammatory, anti-stress, antioxidant, and antiviral		
Conservation status	Appendix II, 2022 (Convention on International Trade in Endangered Species CITES, 2024); Global G5 Secure status (NatureServe Explorer, 2023b) The IUCN Red List of Threatened Species: Least Concern, 2014		
Habitat/habitat loss	Reproductive success	Climate change	Bio-economy (use)
In principle, this is a very widely distributed species in both North America and Eurasia (György et al., 2018) *R. rosea* typically grows in cold regions such as the Arctic and mountainous regions of Europe and Asia (Kubentayev et al., 2021; Prokopyev et al., 2021; Small and Catling, 1999). Its preferred habitats are cool, moist, and well-drained environments (Terletskaya et al., 2023). Changes in vegetation composition due to climate change and human activity can alter the ecological balance, making habitats less suitable for *R. rosea* (You et al., 2018a). Increased competition from other species and the introduction of invasive species also pose threats	*R. rosea* is a slow-growing plant with specific ecological requirements, making it less competitive compared to weedier species (Terletskaya et al., 2023). Its reproductive success is closely tied to environmental stability, and it does not readily invade disturbed areas	Changing temperature and precipitation patterns pose a threat to the species by altering environmental conditions, potentially reducing suitable habitat. Projected data for the Altai Mountains (Prokopyev et al., 2021) and Tibetan Plateau (Yang et al., 2023; CITES, 2023), namely, increasing temperatures and changing precipitation patterns, point to a potential effect on the distribution and abundance of *R. rosea*. These changes may lead to shifts in its geographic range, potentially reducing its available habitat	Wild harvest yield: 100–500 kg of dried root/ha/year. Cultivated yield: 1,000–2,000 kg of fresh root/ha/year = 200–400 kg of dried root/ha/year (Galambosi et al., 2007; Galambosi et al., 2006). Overharvesting, mining activities, and agricultural expansion are significant threats to the natural habitats of *R. rosea*. These activities lead to habitat degradation and fragmentation, reducing the species’ viable living areas. Changes in vegetation composition due to climate change and human activity can alter the ecological balance, making habitats less suitable for *R. rosea*. Increased competition from other species and the introduction of invasive species also pose threats
	Limited genetic diversity in certain populations, due to overharvesting and habitat fragmentation, increases the species’ vulnerability to environmental changes and diseases		

Assessment: Overharvesting poses a significant threat to wild populations, necessitating sustainable practices and regulations. Climate change has a significant effect on the species’ reproductive capacity, leading to a reduction in its populations in natural conditions. Certification programs like FairWild (ARRGO, 2021; FairWild Foundation, 2023) promote responsible harvesting to balance market demand with conservation efforts. Ensuring sustainable harvesting is crucial for the long-term preservation of *R. rosea*.

**TABLE 8:** *Warburgia salutaris* (G.Bertol.) Chiov., as a case study for vulnerable species in southern Africa; for the three levels of concern, see Table 1.

Species name	*Warburgia salutaris* (G.Bertol.) Chiov., Cannellaceae; pepper-bark tree		
Botanical drug use	Bark, to a lesser extent leaves and root bark		
Use in medicine	Respiratory tract infections, gastrointestinal ailments, inflammation, pain and skin conditions		
Conservation status	Vulnerable C2a(i), from 2022		
Habitat	Reproductive success	Climate change	Bio-economy (use)
The species occurs in South Africa, Eswatini (previously Swaziland), Lesotho, Zimbabwe (Mutema Highlands) and Mozambique (Maputo Province) (Harvey-Brown et al., 2022). The tree grows in montane forests and evergreen forests along the coast (Maroyi, 2013) as well as bushveld areas (Senkoro et al., 2019)	Propagation from seed is difficult with extensive parasitism noted in seeds and difficulty with storage (Symmonds and Crouch, 2000; Botha et al., 2004). Most populations have been shown to reproduce clonally (Botha et al., 2004). Shoot-tip cuttings are used in cultivation (Symmonds and Crouch, 2000) and a tissue culture technique is available (Kowalski and van Staden, 2001)	Climatic model predictions suggest that the distribution of *W. salutaris* is not likely to decline because of factors related to climate change (Senkoro et al., 2024). However, this study did not account for the effect of climate change on species interacting with *W. salutaris*, including humans, pollinators, and parasites	Cunningham (Cunningham, 1988) estimated that 315 bags of bark were traded between 54 herb traders annually in KwaZulu-Natal in 1988. In 1998, Mander (Mander, 1998) estimated annual trade volumes of 17.2 tonnes of *W. salutaris* bark in the same province. The pressure on the species has been exacerbated by habitat degradation and transformation (Veeman et al., 2014; Cunningham, 1993). Many medicinal plant harvesters in South Africa travel from outside the harvesting areas, collecting plant material in bulk to supply urban traders. In 2020, Senkoro et al. (2020) reported that 43% of *W. salutaris* bark in the major market in Johannesburg (South Africa) was sourced from Mozambique, with annual amounts traded of 500–1,000 kg
	Overharvesting can damage the tree, reducing its lifespan		

Assessment: *W*. *salutaris* is threatened by overharvesting and habitat loss. It is readily cultivated, but current levels of cultivation are insufficient to meet demand. With the time taken for trees to grow large enough to be harvested, it is essential to carefully manage wild populations.

### 2.1 Case study 1: *Aquilaria malaccensis* Lam

*A*. *malaccensis* is widely distributed in Bangladesh, Bhutan, India, Indonesia, Malaysia, Myanmar, Nepal, Philippines, Singapore, and Thailand (Oldfield et al., 1998). It is now listed as critically endangered by the IUCN in all those countries (IUCN, 2024). *A. malaccensis* produces a highly valuable fragrant resin that impregnates the heartwood (agarwood) in response to fungal and other microbial infections (Chhipa and Kaushik, 2017).

The wood has been traded globally for centuries and is deeply rooted in some religions’ spiritual culture. Beyond its cultural and religious uses, it is also traditionally used for health problems, such as skin problems, thyroid problems, and joint pain (Grosvenor et al., 1995). The versatile uses of agarwood have led to a global increase in demand, endangering the natural populations of *A. malaccensis*. Overharvesting and unsustainable harvesting techniques are the main problems causing the decline of wild populations. In the past, collectors harvested agarwood from the forest by cutting dozens of *Aquilaria* trees because they could not accurately identify which trees contained resin and were ready to be harvested (Irnayuli et al., 2011).

Regarding production, Indonesia, Malaysia, and Thailand are the largest exporting countries in South Asia. From 2000 to 2023, these countries exported 2.448,5, 2.158,5, and 1.827,6 metric tons of agarwood chips, respectively. In the case of Indonesia and Malaysia, practically all material is collected from the wild (99,6%). Thailand and Malaysia are the biggest exporters of essential oil products, with around 4,2 and 1.5 metric tons from 2000 to 2023. On the other hand, around 57% of Thailand’s oil production comes from plantation agarwood of three species, whereas only 19% is produced in Malaysia (Thompson et al., 2022).

It is estimated that more than 60 million trees have been planted (IUCN, 2024) and resin induction techniques have been developed to meet high market demand and maintain the sustainability of wild plant habitats (Thompson et al., 2022). In contrast, the trees are found in low densities (up to 2/ha) in the wild, and only a small percentage of all non-cultivated trees–between 1% and 10% – produce agarwood with valuable resin (Thompson et al., 2022). Artificial methods for inducing infection have been developed to produce the resin using highly virulent fungi like *Fusarium* spp. This method has successfully increased the relative share in exports of products from cultivation compared to wild-harvested products (Thompson et al., 2022). However, the increase in the ratio does not necessarily prevent a decline in wild populations. Illegal harvesting still occurs in almost all producing countries because the agarwood harvested from wild *Aquilaria* is generally considered higher quality than cultivated *Aquilaria* (Kanazawa, 2017; Tamuli et al., 2005).

Another strategy to address overexploitation is logging restrictions and prohibitions for the species. Wild *Aquilaria* logging is illegal in several countries, with only Indonesia and Malaysia still allowing it under quotas and controls. China, Malaysia, Nepal, and Indonesia use assisted natural regeneration to rebuild wild populations. Only oil and live plants are subject to export licenses in India, while raw materials are unavailable (Thompson et al., 2022).

Although the survival and habitat of *A. malaccensis* seem to be impacted by climate change, it depends mainly on annual average rainfall, soil pH, and yearly average temperature. Changes in temperature, rainfall, and precipitation associated with climate change will affect the survival of this species. Tropical plants are predicted to face the most significant risk from climate warming as they experience temperatures closer to their upper germination limits (Sentinella et al., 2020). This is supported by evidence that recent droughts in parts of Southeast Asia (e.g., Thailand and Myanmar) have resulted in high agarwood tree mortality in some plantations (Thompson et al., 2022). Moreover, pests and diseases also threaten the life of this species. In several cultivation sites in India, most plantations face high mortality rates due to dry soil, moist weather, and high temperatures (Bhattacharjee et al., 2024). Furthermore, sap-suckers, leaf defoliators, and wood borers (insects from the orders Coleoptera and Lepidoptera), and several diseases are known to damage leaves, stems, and roots, especially in young plants (Samsuddin et al., 2019). Overharvesting and unsustainable harvesting are the main problems with this species’ availability in nature. Changes in temperature and rainfall correlated with climate change can also change the distribution and population, accelerating the decline of the wild population (Table 2).

### 2.2 Case study 2: *Boswellia sacra* Flück

*B. sacra* trees and other species produce an aromatic resin known as frankincense, widely used in religious rituals, perfumes, and as an ingredient in traditional medicines. Frankincense has a known medical and religious history dating back nearly 5,000 years (DeCarlo et al., 2023). This resin has been used to treat wounds, skin infections, inflammatory diseases, dementia, and other ailments, in medicine systems including Ayurvedic and TCM. Its widespread use over thousands of years makes it one of the oldest globally traded commodities (DeCarlo et al., 2020; Khalifa et al., 2023).

*B. sacra* is native to the arid regions of Oman, Yemen, Ethiopia, Somalia, and Kenya (Hamdiah et al., 2022; Eshete et al., 2012; DeCarlo et al., 2023). Harsh climatic conditions, including high temperatures and low rainfall, are the characteristics of these areas. Climate change poses an additional threat to this species, as rising temperatures and changes in precipitation patterns may affect its growth and resin production (Mwijuke, 2024; DeCarlo et al., 2020; Eshete et al., 2012; Gonzalez, 2020). Climate models predict increased temperatures and decreased precipitation in the growing regions of *B. sacra* and *Boswellia papyrifera* (Rajpoot et al., 2020; Gidey et al., 2020). This may lead to habitat loss and reduced species viability. Other factors influencing the decline of tree populations include fires, animal grasing, changes in land use, insect attacks, and improper harvesting (DeCarlo et al., 2020). In Ethiopia, the world’s largest exporter of frankincense, the *Boswellia* tree is predicted to become almost extinct within the next 3 decades (Johnson et al., 2022; DeCarlo et al., 2020).

Trade in frankincense has increased significantly in recent decades due to high demand in global markets. However, this increased demand has led to overexploitation, threatening the sustainability of the species (Gonzalez, 2020). In impoverished regions of Somali, cutting down and collecting is a primary and crucial source of income for the deprived populated areas (DeCarlo et al., 2020). The problem is aggravated by the fact that *Boswellia* trees have a limited growing range at high altitudes with specific climates and growing conditions in the arid regions of the Middle East (Gonzalez, 2020). These factors make the survival of frankincense even more difficult.

*B. sacra* produces a significant number of seeds, but the viability of these seeds varies greatly depending on the health of the parent trees and environmental stressors, and germination rates under natural conditions are very low, typically between 1% and 10% (Hamdiah et al., 2022; Al-Harrasi et al., 2019). This low rate is attributed to various factors, including issues with genetic diversity, environmental conditions, and seed viability. Attempts to improve germination rates have been made using a variety of methods. For example, seed sorting by flotation can help remove non-viable seeds, and specific growing conditions such as optimal moisture, temperature, and light are critical to improving germination success. Some studies have shown that controlled pollination and selective breeding can achieve higher germination rates, sometimes reaching 40%–60% under ideal conditions, but more research is needed. DeCarlo and co-authors (DeCarlo et al., 2020) highlights the discrepancy between production and trade volumes. This indicates that current harvesting practices are unsustainable, and that overharvesting reduces resin quality and quantity. *B. sacra* and related species face multiple threats from overexploitation, climate change, habitat loss, and biotic determinants. It is crucial to implement sustainable harvesting practices, protect its habitat, and involve local communities in conservation efforts (Table 3).

### 2.3 Case study 3: *Crocus sativus* L.

Saffron is known for its high price and complex technological process of growing and processing. *C. sativus*, from which saffron is obtained, depends on human propagation (El Merzougui et al., 2024; Busconi et al., 2018). According to some sources, its wild forms were found only on the islands of Crete and Santorini in 2,000–1,800 BC, that is, 3,800–4,000 years ago (Nemati et al., 2019). Since then, the species has propagated vegetatively only with human participation (Busconi et al., 2018). Traditionally, *C. sativus* is grown in large quantities in Iran, India, Morocco, the United States, Spain, and Greece. However, *Crocus* is also cultivated in other European countries, although in smaller volumes (Kothari et al., 2021; Mehmeti et al., 2024).

Saffron is considered one of the most ancient products in the world–it is mentioned in Egyptian papyri, the works of Homer and Hippocrates, and even in the Bible, along with olives and figs (Hofmann and Ratsch, 2005). It is the most expensive spice in the world; its price can range, depending on quality, from USD 5 to 11,000 per 1 kg (Mehmeti et al., 2024). Saffron has a variety of uses, from cooking (Butnariu et al., 2022) to traditional medicine, for its anti-cancer (Bhandari, 2015) and neuroprotective properties (Bian et al., 2020; Abdian et al., 2024). Saffron is described in several World Pharmacopoeias (Mykhailenko et al., 2022; Mykhailenko et al., 2020a), but it remains a poorly recognised and appreciated medicinal plant in mainstream medicine. Demand for the spice is steadily growing, while recently, there has been a significant decrease in harvest volumes due to geographical and climatic conditions (Pirasteh-Anosheh et al., 2023; Razmavaran et al., 2024). Farmers have repeatedly experienced significant climate impacts on shifting planting and harvest dates, as well as reducing harvest volumes and the quality of plant materials (Sam, 2024).

The annual global production of saffron is estimated to be around 300 tons, with the main producers being Iran (90%–93% of global production), India, Greece, Morocco, and Spain (Busconi et al., 2018; Fernández et al., 2011). During 2023–2024, Iran saw a sharp decline in saffron production linked to several causes: climate change (average temperature increase of almost 2.8°C in October and November 2022 compared to 2021), poor agricultural practices, lack of rainfall (down 52% in the last winter of 2023 and during flowering), drought in certain areas (like Gonabad, Mah-Velat and Khaf), unusually cold winters and warm weather during saffron flowering (SunLand Saffron, 2024). In most regions, saffron production in Iran has decreased by more than 60% in the last 50 years. According to the Khorasan Razavi Agricultural Organization, Iranian farms have produced an average of 350–370 tons of saffron in recent years, while only 140 tons of saffron were produced in 2023 (SunLand Saffron, 2024).

Similar changes have occurred in Kashmir (India), the second-largest saffron-producing region and a traditional growing area since the 5th century BC (Desk, 2024; Bukhari, 2020; Saraf et al., 2018). Due to distinct climate changes, drought conditions in the region have meant that saffron yields have halved over the past 2 decades. There has been a decline in production in Kashmir since 1999 due to decreased rainfall, delayed flowering, and impact on crop yields (Husaini, 2014); productivity reduced in 1999–2003 from 3.12 to 1.57 kg/ha. In a report on the harvest in November 2023, Indian media outlet News18 reported that the region now produces an average of 2.34 tons of saffron per year, or less than a quarter of its production over 6 years ago. In February 2024, Agriculture and Farmers Welfare Minister Arjun Munda said that saffron production in Kashmir decreased by 67.5% between 2010 and 2023, but from 2022–2023, saffron production increased marginally by 4% (Aamir, 2024). In 1930, Spain was the world’s largest saffron producer, harvesting 120 tons of saffron grown on 13,000 ha of land annually (SunLand Saffron and Herbs, 2022). By the 1970s, annual production had dropped to 70 tons. Today, about 140 ha, the vast majority of them in Castilla-La Mancha, are devoted to *C. sativus*. Total production for 2022 was approximately 450 kg. Today, Iran produces between 200 and 250 tons of saffron per year, and Greece produces about 2.5 tons (Sam, 2024).

Currently, there is no stable production of saffron in European countries since the corm is susceptible to fungi and very sensitive to temperature changes. In recent years, 200 producers whose saffron origin status is protected have seen their crops affected by the climate emergency. Saffron cultivation is highly dependent on specific climatic conditions. Unpredictable weather patterns, such as temperature fluctuations and irregular rainfall, can impact on saffron yields, posing a challenge for consistent production (Table 4).

### 2.4 Case study 4: *Panax quinquefolius* L.

Ginseng root in commerce is obtained from diverse species of *Panax* (Araliaceae), including *P. ginseng* C.A.Mey. (Korean or Chinese ginseng). *P. quinquefolius* as well as *Eleutherococcus senticosus* (Rupr. & Maxim.) Maxim. (Siberian ginseng, Araliaceae). In terms of sustainability, there is a similarity in its use and pharmacological profile to the Asian species of *Panax*. American ginseng and Asian ginseng may increase energy levels, lower blood sugar and cholesterol levels, relieve stress, promote relaxation, treat diabetes, and control sexual dysfunction in men (Brinckmann and Huang, 2018).

Despite the widespread and stable cultivation of *P. quinquefolius* in Canada (60%), the United States (30%), and China (7%) (Shen et al., 2019; Xiao, 2000), interest in American ginseng wild-harvested raw materials from North America (United States: Connecticut, Maine, Massachusetts, New Hampshire, Rhode Island, Vermont, and Missouri) and Canada still exists. Vandalism and illegal harvesting of raw materials in forests continue despite the existing status of the species as being endangered nationally [Vulnerable (G3)] and laws for its protection and conservation, especially in Ontario and Quebec (Government of Canada, 2015). American ginseng is now considered an endangered species in 16 United States states, and in another 10 states of the United States, *P. quinquefolius* is listed on Appendix II of CITES (Convention on International Trade in Endangered Species CITES, 2024), which restricts trade in whole roots and root slices.

The uncontrolled digging of wild roots for export is due to the high price of raw materials on the black market based on its popularity in traditional Chinese medicine (White, 2000). Another threat is the logging of mesic hardwood forests since the species requires rich soil in a moist, generally shaded setting. Ginseng is physiologically adapted to low light levels and can experience early leaf senescence or depressed growth with moderate-high light levels (USFWS, 2005), which can be an issue with increased forest fragmentation.

Sales of wild and cultivated ginseng exceed USD 25 million each year in North America, with wild ginseng seen as the more desirable type. Data from the U.S. Fish and Wildlife Service indicate that thousands of pounds of wild ginseng roots are exported annually. For instance, in 2019, over 80,000 pounds of wild ginseng were exported from the United States. Since the process of plant cultivation faces various problems, the increased interest in wild forms of the species is due to environmental changes that reduce the cultivation area and the quality of the product itself. Therefore, for the introduction and cultivation of plants, it is necessary to identify the maximum similarity of the new ecological distribution of *P. quinquefolius* and predict its response to future climate change. Various simulations of American ginseng responding to a 1°C increase in maximum growing season temperature over the next 70 years showed that the risk of local extinction for average population size (*n* = 140) was 65%, which far exceeds the additive effect of the two factors (risk of extinction = 8 and 6% for deforestation and climate change respectively) (Souther and McGraw, 2014). Among the main external factors of plant mortality are the effects of warming and illegal harvesting, leading to the loss of natural habitat, such as deforestation (Souther, 2011; Souther and McGraw, 2014; Souther et al., 2012). Internal reasons for decreased yields of American ginseng include slower plant growth, reduced fertility, and disease susceptibility.

Additionally, factors affecting plant populations also include predatory wild animals eating young plants, which affects the growth and survival of ginseng, as well as the influence of invasive species competing for habitat (McGraw et al., 2013). However, the key and most dangerous influence is human activity (Case et al., 2007), which, on the one hand, leads to unsustainable harvesting methods or urbanisation of the territory, which leads to habitat destruction, and on the other hand, indirectly affects the intensity of climate change and its impact on a species. Changes in temperature and precipitation patterns due to climate change could affect the distribution and health of ginseng populations (Canada. Environment and Climate Change Canada, 2018). Increasing temperatures and changes in precipitation lead to habitat loss and increased vulnerability to pests and diseases. Regional studies indicate that North-eastern U.S. forests may face increased temperatures and altered precipitation, potentially stressing ginseng habitats (McGraw et al., 2013) (Table 5).

### 2.5 Case study 5: *Pilocarpus microphyllus* Stapf ex Wardlew

*P. microphyllus* (or jaborandi) is widely distributed in the northern region of Brazil (Sawaya et al., 2011) and is one of the most important commercial products of the native Brazilian flora. The genus *Pilocarpus* is the only natural and economically viable source of pilocarpine currently known (De Abreu et al., 2005). Four (of 17) species, including *P. microphyllus* (also *P. alatus, P. carajensis,* and *P. sulcatus)* have been listed as threatened in Brazil due to a lack of sustainable management (Abreu et al., 2007). Pilocarpine, an important imidazole alkaloid, is extracted from the leaves of jaborandi, and is a sympathomimetic agonist in ophthalmology, where it is used as a miotic, in open-angle glaucoma, and to contract the pupil after the use of atropine (Sneader, 2005). Pilocarpine is also a powerful stimulant of salivation and perspiration (Budavari, 1989). In 1994, pilocarpine was approved by the North American Food and Drug Administration (FDA) for the treatment of post-radiation xerostomia (dry mouth) in patients with head and neck cancer (Gornitsky et al., 2004; Sidhu, 2014). Scientific studies have reported that the pilocarpine content in jaborandi varies in response to abiotic determinants and seasonally (Avancini et al., 2003; Monteiro et al., 2023). Plants of jaborandi in the forest seem to accumulate more pilocarpine in the leaves, with mean values ranging from 400 to 500 mg/g (Sousa, 1991).

The German pharmaceutical company “Merck” has held a decade’s monopoly on the purchase of jaborandi leaves and the production of pilocarpine in Brazil, most importantly in Maranhão, which produces around 95% of all national production (IBGE 1975–1998) (Pinheiro, 1997). These approximately 2,300 ha have partially replaced the extraction process of wild populations. The main destinations of pilocarpine for international trade are Germany and the USA, with around 70% and 17% of Brazilian exports, respectively (CNI, 2014). In Germany, the “Boehringer-Ingelheim” company has a virtual monopoly on the distribution of pilocarpine (CNI, 2014). The price paid by pharmaceutical industries prompted the widespread participation of local people in collecting the leaves of bushes growing in the forests. As a consequence of this intense gathering, jaborandi was included in the Brazilian list of endangered species with a status vulnerable A2cd (Pinheiro, 1997; Pinheiro, 2002).

This shrub is native but not endemic to northern and northeastern Brazil, occurring more specifically in eastern Pará, northwestern Maranhão, and northern Piaui and to some areas in French Guiana and Suriname (Pirani and Groppo, 2014). This region is characterised by annual precipitation between 2,000–2,400 mm, while the average annual temperature varies from 23°C to 26°C (Moreira et al., 2021), which offers the species the maturation of its fruits and flowers and, subsequently, good growth. The natural habitat is open sunny forest habitats, river basins with sandy soils, and rocky outcrops of pre-Amazonian forests (Skorupa, 2000). The main threat to this species’ habitat is anthropogenic activity, such as Amazon deforestation for agriculture and mining. Being a hermaphrodite, cross-pollination is the norm. Unfortunately, no information is available regarding this species’ reproductive method (Sandhu et al., 2006). Recent studies indicate that this species has high levels of genetic diversity and an effective population size sufficient to reduce the probability of extinction due to inbreeding depression (Monteiro et al., 2022).

The investments and technological advances advocated to increase productivity and leaf yields indicate that pilocarpine has long been a profitable business for the pharmaceutical industry, as well as supporting the development of agriculture and other forms of harvesting raw materials among local people (Pinheiro et al., 2006). The development of technologies that allowed for the cultivation of jaborandi in small regions could serve as a method for reducing pressure on natural populations (Caldeira et al., 2017). Propagation of these species is usually carried out by producing seedlings from recently collected seeds, allowing germination to reach up to 96% (de Meneses et al., 2007), with peak fruiting and seed dispersal occurring from May to July under natural conditions (Muniz, 2008; Rocha et al., 2014). Conservation programs and management plans have been established for this species in the Carajás National Reserve (Pará, Brazil) (Monteiro et al., 2022). Sustainable management in natural areas and cultivation are potential avenues for plant conservation. The development of a germplasm bank and rescue accessions are solutions that can be utilised to preserve genetic material for breeding, involving the reintroduction of thousands of plants in areas designated for conservation and sustainable uses. To accomplish this, a series of multidisciplinary studies will be required, including ones on genotype selection, propagation, maximising biomass and pilocarpine production, and identifying nutritional requirements and environmental variables that will boost pilocarpine yield (Caldeira et al., 2017) (Table 6).

### 2.6 Case study 6: *Rhodiola rosea* L.

*R. rosea*, commonly known as rose root, is a perennial species known for its adaptogenic properties for stress and fatigue, which make it popular in traditional medicine and modern supplements, especially in Russia, Scandinavia, and China (Aiken et al., 2007; Booker et al., 2016). However, unlike *C. sativus* and *P. quinquefolius*, in addition to the significant exploitation of the species, another problem is climate change, which leads to the migration of plant populations to colder zones of the Alps (You et al., 2018b). The global market for *R. rosea* is estimated at tens of millions of dollars per year (360ResearchReports, 2022). The species is collected from wild populations and cultivated sources, but the collection of wild plants predominates due to the perceived higher potency of wild accessions. Overharvesting of wild populations, especially in Russia, Mongolia, and China, has led to significant declines in natural populations. Sustainable harvesting methods are not widely adopted, leading to the risk of local extinction and genetic depletion.

*R. rosea* usually grows in cold, moist, and well-drained environments such as the Arctic, North America, Canada, and mountainous areas of Europe and Asia (Small and Catling, 1999; Brown et al., 2002; Hung et al., 2011), which are especially vulnerable to temperature shifts caused by climate change. Recent studies have shown that *Rhodiola* is sensitive to changes in temperature and rainfall patterns (You et al., 2018b). Climate change poses a threat by altering environmental conditions and potentially reducing suitable habitats. In regions such as the Altai Mountains and the Tibetan Plateau, rising temperatures and changing rainfall patterns are expected to affect the distribution and abundance of *R. rosea* (Kubentayev et al., 2021; Klanderud, 2005). These changes could alter its geographic range, potentially reducing its available habitat.

Biotic determinants affecting the specie' abundance include slow growth rates, special habitat requirements, and limited seed dispersal, which contribute to its vulnerability. However, habitat loss due to forced migration is the main threat to the species, caused by both natural and anthropogenic determinants. Over the last decade, the populations have decreased significantly, and the species has the status of “endangered” and is listed in Appendix II (CITES). In addition, *R. rosea* also has Global G5 Secure status (from 2015) in Canada and the U.S. (NatureServe Explorer, 2023b). Promoting the cultivation of *R. rosea* as an alternative to wild collection can help meet market demand while conserving wild populations. Research into effective cultivation methods continues and has shown promising results in reducing pressure on natural habitats (Table 7).

### 2.7 Case study 7: *Warburgia salutaris* (G.Bertol.) Chiov.

*W. salutaris* (known as pepper-bark tree, referring to the peppery taste of the bark and leaves) is an endangered species highly valued in South African traditional medicine (Williams et al., 2013; Van den Bosch et al., 2023). The bark of the tree is used to treat a multitude of ailments, including coughs, fever, colds, headaches, inflammation, bladder infections, abdominal pains, skin irritations, ulcers, and sores (Maroyi, 2013; Van Wyk and Gericke, 2000; Van Wyk et al., 2009). Leaves and root bark are also used but to a much lesser extent. The plant material is traded commercially at traditional medicine “*muthi”* markets, resulting in unsustainable harvesting of wild populations (Williams et al., 2013; Burrows et al., 2018). The leaves and bark both contain drimane sesquiterpenoids (Mashimbye et al., 1999), such as polygodial and isopolygodial, warburganal, salutarisolide, mukaadial, and muzigadial (Rabe and van Staden, 2000).

*W. salutaris* is capable of regenerating vegetatively by suckering, but a loss of sexual reproduction will result in decreased genetic diversity in a population, negatively affecting long-term persistence (Botha et al., 2004; Johnson et al., 1995). Clusters of trees studied in KwaZulu-Natal were reported to be generally all clones, with no seedlings found (Scott-Shaw, 2001). Flowers are bisexual but it is unknown whether this species is self-compatible and able to undergo successful self-pollination (Van den Bosch et al., 2023). Outcrossing populations should, in principle, have increased genetic diversity and therefore a greater ability to adapt. *W*. *salutaris* is challenging to propagate from seed owing to a paucity of viable seeds owing to high predation levels, insect infestations, and loss of viability during storage of the recalcitrant, desiccation-sensitive seeds (Symmonds and Crouch, 2000). High phenolic content has impeded tissue culture, but an *in vitro* technique is available (Kowalski and van Staden, 2001). Shoot-tip cuttings have proven successful in cultivation projects (Symmonds and Crouch, 2000).

The population size of *W. salutaris* has been approximated at between 4,638 and 4,566 plants, with the largest recorded subpopulation within the South African range consisting of 952 plants (Harvey-Brown et al., 2022). There are potentially larger subpopulations as yet unrecorded, but these are not likely to have more than 5,000 mature individuals. Conservation of *W. salutaris* was initiated by the company HL&H (now known as Mondi Forests), with the establishment of a living gene bank of about 1,000 trees (Van Wyk, 2011). A significant number of trees was propagated by this organisation for distribution in 1996 when *W. salutaris* was selected as “Tree of the Year” in South Africa. In the Kruger National Park, thousands of cultivated saplings have been distributed to traditional healers, decreasing the rate of decline in the population and improving the species’ status from endangered to vulnerable but declining (Harvey-Brown et al., 2022).

Another conservation initiative is to promote the use of leaves as a substitute for the bark material, as two of the most bioactive ingredients, polygodial and warburganal, have been detected in both leaf and bark extracts (Drewes et al., 2001). Traditional healers occasionally use leaves in treating particular complaints, but if this could be encouraged on a larger scale, more sustainable harvesting levels could be achieved from wild as well as cultivated populations (Botha et al., 2004). However, Leonard and Viljoen (Leonard and Viljoen, 2015) cautioned that the shorter shelf-life of leaves compared to that of bark may be a concern for traditional healers.

The most significant threat to *W. salutaris* is the overharvesting of bark and roots for traditional medicinal use (Veeman et al., 2014). The tree can tolerate some harvesting by producing coppice shoots and regrowing stripped bark, but excessive harvesting can cause the death of the tree (Harvey-Brown et al., 2022). Overuse of the bark has resulted in the species being listed as extinct in the wild in Zimbabwe (Maroyi, 2008) and locally in some regions of KwaZulu-Natal, South Africa (Harvey-Brown et al., 2022). The mean thickness of bark sold in the Johannesburg markets decreased significantly from 1994 to 2001, indicating that bark from smaller trees was increasingly harvested as larger, more mature trees became unavailable (Williams et al., 2007). Other factors that influence tree growth include agricultural activities, the use of trees as building material, road construction (Harvey-Brown et al., 2022; Veeman et al., 2014; Cunningham, 1993), fires (Senkoro et al., 2020), grazing by herbivores, termite infestation, and damage caused by fungal diseases (Harvey-Brown et al., 2022).

Information on the environmental requirements and precise distribution of *W. salutaris* is scanty. The distribution of *W. salutaris* is dispersed and fragmented at present, possibly as a result of biogeographical and climatic influences, but its ecological requirements are largely unknown (Senkoro et al., 2024). In an earlier study, it was suggested that the species is sensitive to lower temperatures and has a reasonable tolerance to drought and precipitation levels, with an affinity for more moist areas (Senkoro, 2021).

Climate projections have predicted an increase in intensity of rainfall over the eastern parts of southern Africa, but that rainfall would be less frequent (Kusangaya et al., 2014). In the Kruger National Park, changes in the intensity and timing of certain environmental conditions, including droughts and floods, were proposed by Kitajima and Fenner (Kitajima et al., 2000) to be detrimental to the establishment of *W. salutaris* seedlings. However, using climatic model predictions, it was reported that for the next 80 years, the distribution of *W. salutaris* is not likely to decline because of factors related to climate change (Senkoro et al., 2024). This study did not take into account the effect of climate change on species interacting with *W. salutaris*, including humans, pollinators, and parasites. Although climate change may not pose the greatest threat to the sustainability of *W. salutaris*, cultivation and reintroduction alone are not likely to enhance the conservation of the species if the threat of overexploitation of bark for medicinal use, as well as the impact of wildfires, charcoal production, and land use transformation, are not addressed. In summary, an integrated management strategy for *W*. *salutaris* is essential to ensure its sustainability. This involves increasing cultivation and redistribution efforts as well as successfully managing protected areas (Table 8).

## 3 General discussion

The species selected for consideration are highly valued for their medicinal, aromatic, or commercial uses, and some could be facing the issues of over-collection, putting their populations at risk of decline (Figure 2). These species are native to specialised or fragile ecosystems that face threats from habitat destruction and climate change (Soehartono and Newton, 2001; Johnson et al., 2022; Kubentayev et al., 2021; Prokopyev et al., 2021; You et al., 2018a; Cunningham et al., 2020; Kauffman, 2006b). As a result, they are classified as vulnerable, endangered, or critically endangered (Convention on International Trade in Endangered Species CITES, 2024; Johnson et al., 2022; Zou et al., 2023; Hazarika et al., 2023), necessitating efforts for their conservation and sustainable use (Tables 2–8). For example, *R. rosea, P. quinquefolius, A. malaccensis, W. salutaris,* and *B. sacra* exhibit low seed viability and slow reproductive success, making natural regeneration difficult. This further increases their vulnerability to environmental changes and diseases (Tables 2, 3, 5, 7, 8).

For example, a significant problem with cultivated *C. sativus* is that it reproduces only through corms rather than seeds, which limits genetic diversity and increases susceptibility to diseases and pests. In addition, this species relies heavily on human intervention for its cultivation. Trials utilising monoculture methods for *C. sativus* further exacerbate vulnerability to environmental changes and diseases. In this case, one actively developed solution is large-scale vertical farming (Nájera et al., 2023; Ambitas, 2024). The same approach is also developed in case of *R. rosea* (Reinhard Bott, Karlruhe, Germany, pers. comm.), which also is characterised by a low reproductive success rate.

In many cases, habitat loss plays an important role. For example, *A. malaccensis* is facing habitat loss due to illegal logging and land conversion, limiting the species’ natural distribution (Tables 2, 4, 5, 7).

Overharvesting and habitat loss pose common challenges for all the species selected in this study (Figure 2). For example, *B. sacra* (native to Somalia and Yemen), *W. salutaris* (found in southern Africa), and *A. malaccensis* (from (Malaysia and Indonesia) face significant threats due to habitat destruction, including deforestation and conversion to agricultural land, as well as overexploitation of their valuable resins (Table 8). Unauthorised overharvesting, vandalism, and habitat loss also severely impact *P. quinquefolius* populations in the USA, *R. rosea* in Kazakhstan and Mongolia, and *P. microphyllus* populations in the Amazon region. Although *C. sativus* is a cultivated plant, its reliance on traditional growing areas limits its distribution and increases the risk of decline. Traditional saffron-growing regions in Kashmir and Iran are experiencing a reduction in arable land due to urbanisation and climate change (Tables 2, 4, 5, 7).

In addition to highlighting the current status or data available for the selected species, the case studies underscore the necessity for stricter regulations across all aspects of trade, from initial harvesting to the availability of products for end users. Within this context, we highlight four key areas of concern (the main determinants of a species’ sustainability) related to the loss of certain species (Figure 1). It is important to note that not all of the above-described determinants are related to climate change and may stem from human activities and/or internal plant characteristics (Pacifici et al., 2015). Climate change adds significant pressure on these species, which requires a better understanding of all factors and their interplay.

Climate change directly impacts on the ability of these species to thrive in their native habitats. Alterations in temperature, rainfall patterns, and soil conditions disrupt the delicate balance required for their growth and survival. Even minor changes in growing seasons can lead to increased susceptibility to diseases, as exemplified in the case study of *P. quinquefolius*. Warmer winters and unpredictable frosts in the Appalachian region affect ginseng growth cycles and increase disease outbreaks. Similarly, *B. sacra* and *A. malaccensis* also show significant sensitivity to fluctuations in temperature and precipitation, which affects resin production in these trees. For *P. microphyllus*, alterations in precipitation and temperature patterns also affect growth and alkaloid production (Tables 2, 3, 6). In Oman and Somalia, changes in precipitation patterns have affected the health and resin yield *of Boswellia* trees, while shifts in climate have similarly affected *Aquilaria* resin in Southeast Asia. *C. sativus* and *R. rosea* are particularly vulnerable to temperature changes, which affect their flowering and growth cycles. Increased temperatures shorten the flowering period of the plant, leading to shifts in planting and harvesting schedules, ultimately reducing yields (Table 9). These challenges highlight the need to consider relocating production zones further north or south to ensure a stable supply (Xu et al., 2019).

**TABLE 9:** Summary of challenges associated with the collection of starting plant materials and their supply for the selected case studies (for references see the relevant tables on the individual case studies).

Species name	Harvest and supply	Challenge	Core needs/alternative strategies
Saffron	The labour-intensive harvesting process increases costs and affects supply consistency	Saffron requires hand-picking of flowers, leading to high labour costs and difficulty scaling production	No clear strategy is feasible aside from increases in the production areas
American Ginseng	High demand and overharvesting threaten sustainable supply	The lucrative nature of ginseng has led to illegal poaching and overharvesting, particularly in national parks	More stringent regulations on harvesting
Rose root	Unsustainable harvesting practices	Increased global demand has led to overharvesting and insufficient time for natural recovery of populations	More stringent regulations on harvesting, and incentives for larger scale agricultural production
Frankincense	Unsustainable tapping practices reduce tree longevity and resin yield	Over-tapping and improper techniques damage trees, leading to reduced resin production and tree mortality	Alternative sources will be key to securing a future for this species, requiring changes to the regulatory framework for commercial products
Agarwood	Illegal trade and overharvesting for perfumery and traditional medicine	The high market value of agarwood has driven illegal harvesting, threatening wild populations	More stringent regulations on harvesting
Jaborandi	Overexploitation for pharmaceutical purposes	Excessive harvesting for the alkaloid pilocarpine, used in glaucoma treatments, threatens natural populations	Expanded silvicultural production, including novel mixed forest production systems
Pepper-bark tree	Habitat loss due to overharvesting and deforestation	Excessive harvesting does not allow the species to recover naturally, often leading to tree mortality	Increased cultivation and protection of wild populations

Unsustainable harvesting practices and high market demand create challenges in supplying medicinal plant material to the pharmaceutical and health food/botanical markets. This requires strategic changes to how relevant industries manage their value chains. For example, the high labour costs associated with the intensive harvesting of *C. sativus* and the illegal poaching of *P. quinquefolius* illustrate the difficulties in maintaining a sustainable supply. The manual picking of saffron flowers and the subsequent separation of the stigma results in high labour costs and problems in the scaling up of production. Furthermore, these plants often do not have sufficient time to regenerate naturally, and improper harvesting practices, particularly concerning resin yield, can lead to plant mortality (Table 9). Sustainable harvesting practices, cultivation, and artificial propagation are being explored to reduce the pressure on wild populations. For example, agarwood plantations are being developed to produce agarwood resin sustainably (Tan et al., 2019), while *P*. *quinquefolius* is cultivated under strict regulations to prevent overharvesting (Burkhart et al., 2021; Burkhart and Jacobson, 2004b; Wang et al., 2024b). Achieving conditions suitable for the species’ natural habitat is essential for ensuring their conservation in the wild (Table 9).

For future research and development, we propose an integrated approach that emphasises the interconnection between research, conservation, cultivation, innovation, regulation, and education (Table 10). This strategy aims to address the critical issues of overexploitation, climate change, and habitat loss that threaten the sustainability of endangered species used in pharmaceuticals. It is based on the framework presented here, which includes seven case studies. In order to structure the strategies for further research and development, we suggest specific actions in six areas of research and development (Table 10), which can form a basis for novel strategies to achieve a more sustainable production of species at risk.

**TABLE 10:** Proposed core themes relevant to an integrated, sustainable approach refocusing on research, development, and use of medicinal/health food plants (MHFPs) based on the assessment of seven exemplary species.

1. Research and Assessment^a^	2. Conservation and Protection^b^	3. Cultivation and Harvesting^c^
• Identify endangered species used in pharmaceuticals	• Implement measures to conserve and protect endangered medicinal species, including establishing protected areas and promoting sustainable harvesting practices	• Promote the cultivation of MHFP through sustainable agricultural practices, such as agroforestry, organic farming, and permaculture
• Analyse the impact of climate change on the distribution and growth of MHFP	• Develop strategies for *ex situ* conservation through botanical gardens, seed banks, and tissue culture techniques	
• Assess current levels of overexploitation and identify regions at risk		• Implement guidelines for sustainable harvesting, including selective harvesting, rotation of harvesting sites, and avoiding collection from sensitive habitats
• Analysis of secondary metabolite diversity in plants and their economic potential	• Engage local communities and indigenous groups in conservation efforts through education and capacity-building initiatives	
• Use of an omic approach for chemical analysis		
• Evaluate environmental factors affecting the growth and sustainability of MHFP.	• Promote collaboration between scientists, pharmaceutical companies, and traditional healers to confirm the effectiveness and safety of herbal medicines	

4. Alternative strategies reducing pressure on species at risk^d^	5. Regulation and Policy^e^	6. Education and Awareness^f^
• Investing in research and development to identify alternative sources of herbal medicines and improve methods of growing MHFP	• Establish regulations and policies to ensure the sustainable management of MHFP resources, including licensing, certification, and enforcement mechanisms	• Raise awareness among healthcare professionals, consumers, and policymakers about the importance of sustainable sourcing and use of herbal medicines
• Research on the potential of biotechnology and genetic engineering to increase the production of desired secondary metabolites in plants	• Incorporate traditional knowledge and practices into legal frameworks to protect indigenous rights and promote cultural heritage	• Provide training and educational programs on sustainable harvesting, cultivation, and conservation practices
• Developing vertical farming approaches for selected species (e.g., *C. sativus*)	• Advocate for international agreements and conventions to address the global trade and conservation of MHFP	• Foster partnerships between academia, industry, and civil society to share knowledge and best practices in sustainable herbal medicines supply chains
• Agroforestry systems for non-timber forest products (e.g., *P. quinquefolius*)		

a Wang et al., 2024a; Husaini, 2014; Souther and McGraw, 2014; Alum, 2024; Mykhailenko et al., 2020b; Pant et al., 2021

b Howes et al., 2020; McLaughlin et al., 2022; Mori et al., 2024; Xu et al., 2019; Groner et al., 2022; Australian Government, 2024; Oldekop et al., 2016; Lee, 2023; Philpott et al., 2022; Asigbaase et al., 2023

c Ncube et al., 2012; Burkhart and Jacobson, 2009; Gamage et al., 2023; European Commision, 2024

d Nájera et al., 2023; Ambitas, 2024; Oldekop et al., 2016; Selwal et al., 2023; Al Aboud, 2024

e Kosoe et al., 2023; The World Health Organization WHO, 1993; Association, 2010

f Booker et al., 2012; Obahiagbon et al., 2023a; Obahiagbon et al., 2023b

A rigorous assessment of sustainability and conservation status (Table 10), in parallel to the safety and efficacy assessment of the plant materials, is essential, particularly for high-value medicinal plants, given the high demand and the potential adulteration in some instances (Booker et al., 2016; Kumar et al., 2023; Püski et al., 2024; Yu et al., 2014). It is essential to remember that the supply of these materials and their release into the pharmaceutical market requires careful strategic planning to ensure ethical sourcing and sustainable practices. Whenever feasible, preference should be given to cultivating endangered species in controlled environments, such as botanical gardens and farms, rather than harvesting from wild populations. This helps conserve biodiversity and supports the implementation of a traceability system that tracks the source of starting plant materials from collection to market, ensuring transparency and compliance with legal and ethical standards.

The approach proposed here is relevant not only in academic contexts but also has the potential to promote the sustainable use of endangered medicinal plants across various industries if these can be produced using more sustainable approaches. Industries can adopt these practices as part of their long-term strategies to ensure sustainability at all levels, including innovative approaches like the “vertical farming” of high-value plants such as saffron, which has demonstrated its potential to generate significant economic benefits (Nájera et al., 2023). Core ecosystem services of forests and non-timber forest products (NTFPs) can be further enhanced if they provide additional income to local communities, as exemplified by successful initiatives in protected areas (Oldekop et al., 2016). These and other strategies (hydroponics, biopriming, biodomes, etc.) can be combined and tailored to specific plant requirements, ensuring the successful cultivation of endangered species even in confined spaces.

While the examples provided illustrate specific problems and potential solutions, many other plant species require our attention and assessment in order to develop appropriate, tailored strategies, including, for instance, *Hydrastis canadensis* L. in North America (Liu et al., 2004), *Arnica montana* L. in Europe (Greinwald et al., 2022), *Nardostachys jatamansi* (D. Don) DC in the Himalayas and Bangladesh (Wen et al., 2022), *Aconitum heterophyllum* Wall. ex Royle in Nepal and Pakistan (Wani et al., 2022), *Angelica sinensis* (Oliv.) Diels in China, North-Central and Mongolia (Deng et al., 2005), *Dendrobium* spp. in Asian countries (Tang et al., 2020), and *Prunus africana* (Hook.f.) Kalkman in Cameroon, Madagascar, Eastern and Southern Africa (Ingram et al., 2015).

## 4 Conclusion

This study provides a framework to address and understand the vulnerability degree (status) of a species based on selected sustainability determinants, including the potential development of approaches to increase the resilience of a species in the context of climate change.

The adaptation and implementation of the proposed framework will require collective efforts and collaboration of key stakeholders. The presented key studies show that each selected species has a different vulnerability assessment of key sustainability indicators depending on the determinant. The analysis also highlights why some species are at a particularly dramatic risk, like *B. sacra*, which is at high risk in all four determinants reviewed. At the same time, climate change in each case is assessed as a risk of extreme vulnerability or destructive indicators.

While the study cannot be comprehensive, it showcases examples of research priorities needed over the coming decades. The presented framework covers a qualitative assessment of species vulnerability and potential actions needed. One can argue that the parameters used here to define vulnerability should be quantified, which may be developed in future studies. However, it also seems problematic since we have a complex network of causal factors, and a quantification can easily lead to misinterpretations. Benchmarking is also an important next step, which will help assess the impact and decide which elements could be quantified (i.e., quantitative assessment).

Here, we argue for a novel, integrated strategy which captures the specific and diverse needs of individual MHFPs. Other case studies following this framework are encouraged, as they may reveal additional aspects of this critical issue. The framework presented here does not claim to have an impact on climate change *per se* or alter policies; however, it lays the scientific foundations necessary to facilitate such changes. This framework highlights the additional benefits of sustainable management and use of medicinal plants. We need a systematic focus on environmental questions, the challenges associated with sustainable production and sourcing, and actions to mitigate or increase resilience in the context of climate change. Given their generally high economic value, medicinal plants offer an opportunity to support *in situ* conservation via financial incentives. For example, it is now well established that secondary forests can capture CO_2_ and thus act as carbon sinks (Heinrich et al., 2023). These core ecosystem services of forests can be enhanced if the forests provide additional income to local communities.

The sustainability of medicinal plants requires a comprehensive and integrated approach. The field of medicinal plant research requires a “paradigm shift” in focus, current strategies, and approaches. By recognising the interactions between natural and anthropogenic determinants, implementing sustainable cultivation practices, and promoting conservation efforts, we can work towards a future where medicinal plants thrive, ecosystems prosper, and human health is preserved. This aligns with the broader challenge outlined by Guerry et al. (2015) as a central task of the 21st century–implementing ‘economic, social, and governance systems capable of ending poverty and achieving sustainable levels of population and consumption while securing the life-support systems underpinning current and future human wellbeing’ (Guerry et al., 2015).

The harmonious coexistence of nature and society depends on our commitment to stewardship, responsible use of resources, and collective efforts to address changing world challenges. This paper contributes to the debate on how this can be achieved and highlights the important role medicinal and high value food plants can play.

## Abbreviations

CITES, Convention on International Trade in Endangered Species; IUCN, International Union for Conservation of Nature; MHFP(s), Medicinal and Health Food Plant(s); NTFP(s), non-timber forest product(s); SDG(s), Sustainable Development Goal(s); TCM, Traditional Chinese medicine.

## References

[B1] 360ResearchReports (2022). *Rhodiola rosea* extract market. Available at: https://www.360researchreports.com/global-and-united-states-rhodiola-rosea-extract-market-20637917. 90

[B2] Aamir A. B. (2024). “Kashmir’s incredible shrinking saffron output,” in Special Features. Available at: https://adnchronicles.org/2024/02/13/kashmirs-incredible-shrinking-saffron-output/.

[B4] Abbass K. Qasim M. Z. Song H. Murshed M. Mahmood H. Younis I. (2022). A review of the global climate change impacts, adaptation, and sustainable mitigation measures. Environ. Sci. Pollut. Res. 29 (28), 42539–42559. 10.1007/s11356-022-19718-6 PMC8978769 35378646

[B241] Abdian S. Fakhri S. Moradi S. Z. Khirehgesh M. R. Echeverría J. (2024). Saffron and its major constituents against neurodegenerative diseases: A mechanistic review. Phytomedicine, 156097. 10.1016/j.phymed.2024.156097 39577115

[B5] Abreu I. N. Mazzafera P. Eberlin M. N. Zullo M. A. Sawaya A. C. (2007). Characterization of the variation in the imidazole alkaloid profile of *Pilocarpus microphyllus* in different seasons and parts of the plant by electrospray ionization mass spectrometry fingerprinting and identification of novel alkaloids by tandem mass spectrometry. Rapid Commun. Mass Spectrom. 21 (7), 1205–1213. 10.1002/rcm.2942 17330216

[B6] Advani N. K. (2023). Assessing species vulnerability to climate change, and implementing practical solutions. Biol. Conserv. 286, 110284–110296. 10.1016/j.biocon.2023.110284

[B7] Aiken S. G. Dallwitz M. J. Consaul L. L. McJannet C. L. Boles R. L. Argus G. W. (2007). Flora of the Canadian Arctic Archipelago. Ottawa: NRC Research Press, National Research Council of Canada. Available at: https://nature.ca/aaflora/data/www/crsero.htm.

[B8] Al Aboud N. M. (2024). Unlocking the genetic potential: strategies for enhancing secondary metabolite biosynthesis in plants. J. Saudi Soc. Agric. Sci. 23 (8), 542–554. 10.1016/j.jssas.2024.06.004

[B9] Alamil J. M. R. Paudel K. R. Chan Y. Xenaki D. Panneerselvam J. Singh S. K. (2022). Rediscovering the Therapeutic potential of agarwood in the management of Chronic inflammatory diseases. Molecules 27 (9), 3038–3060. 10.3390/molecules27093038 35566388 PMC9104417

[B10] Al-Harrasi A. Khan A. L. Asaf S. Al-Rawahi A. (2019). “Propagation and conservation of *Boswellia sacra* ,” in Biology of genus *Boswellia* (Cham: Springer International Publishing), 71–84. Chapter 5.

[B11] Ali T. Deng Q. Zhu A. Xie W. (2024). A framework for analyzing climate change impacts on agricultural value chain. Energy Clim. Manag., 1–11. 10.26599/ecm.2024.9400005

[B12] Alum E. U. (2024). Climate change and its impact on the bioactive compound profile of medicinal plants: implications for global health. Plant Signal. Behav. 19 (1), 2419683–2419686. 10.1080/15592324.2024.2419683 39460932 PMC11520564

[B13] Amaral G. C. Pezzopane J. E. M. de Souza Nóia Júnior R. Martínez M. F. Fonseca M. D. S. Gibson E. L. (2022). *Pilocarpus microphyllus* seedling growth threatened by climate change: an ecophysiological approach. Theor. Appl. Climatol. 147 (1), 347–361. 10.1007/s00704-021-03831-6

[B14] Ambitas (2024). Vertical farm 2.0. Available at: https://www.ambitas.org/solutions/vertical-farming/case-study/vertical-farm-202024.

[B15] Amri M. A. Shanfari A. A. (2024). Harvesting and agro-ecological zones effects on sustainability of *Boswellia sacra* in Oman. Res. Square. 10.21203/rs.3.rs-5222978/v1

[B16] Anaise Costa C. Cristina L. Luana dos Santos S. Vanessa S. S. (1970). Viabilidade de sementes armazenadas de frutos imaturos de jaborandi (*Pilocarpus pennatifolius* Lem. - Rutaceae). Pesqui. Agropecuária Gaúcha 14 (1), 63–66.

[B17] Applequist W. L. Brinckmann J. A. Cunningham A. B. Hart R. E. Heinrich M. Katerere D. R. (2020). Scientistsʼ warning on climate change and medicinal plants. Planta Medica 86 (01), 10–18. 10.1055/a-1041-3406 31731314

[B18] ARRGO (2021). FairWild Week and wildharvested Rhodiola rosea. Thorsby, AB, Canada: Alberta Rhodiola Rosea Growers Organization. Available at: https://arrgo.ca/new2022/2021/06/19/fairwild-week-and-wildharvested-rhodiola-rosea/.

[B19] Asigbaase M. Adusu D. Anaba L. Abugre S. Kang-Milung S. Acheamfour S. A. (2023). Conservation and economic benefits of medicinal plants: insights from forest-fringe communities of Southwestern Ghana. Trees, For. People 14, 100462–100524. 10.1016/j.tfp.2023.100462

[B20] Association (2010). Guidelines for good agricultural and wild collection practices for medicinal and aromatic plants (GACP ‐ MAP). Brussels: European Herb Growers Association, 13.

[B21] Atanasov A. G. Waltenberger B. Pferschy-Wenzig E. M. Linder T. Wawrosch C. Uhrin P. (2015). Discovery and resupply of pharmacologically active plant-derived natural products: a review. Biotechnol. Adv. 33 (8), 1582–1614. 10.1016/j.biotechadv.2015.08.001 26281720 PMC4748402

[B22] Australian Government (2024). Environmental protection and biodiversity conservation act 1999 (EPBC act). Available at: https://www.dcceew.gov.au/environment/epbc.

[B23] Avancini G. Abreu I. N. Saldaña M. D. Mohamed R. S. Mazzafera P. (2003). Induction of pilocarpine formation in jaborandi leaves by salicylic acid and methyljasmonate. Phytochemistry 63 (2), 171–175. 10.1016/s0031-9422(03)00102-x 12711138

[B24] Barcaccia G. Arzenton F. Sharbel T. F. Varotto S. Parrini P. Lucchin M. (2006). Genetic diversity and reproductive biology in ecotypes of the facultative apomict *Hypericum perforatum* L. Heredity 96 (4), 322–334. 10.1038/sj.hdy.6800808 16508660

[B25] Baych A. (2022). “Report Name: American ginseng under review for food ingredient nse. Report number: CH2022-0053,”. Washington, DC: USDA Foreign Agricultural Service, 4. Available at: https://apps.fas.usda.gov/newgainapi/api/Report/DownloadReportByFileName?fileName=American%20Ginseng%20Under%20Review%20for%20Food%20Ingredient%20Use_Beijing_China%20-%20People%27s%20Republic%20of_CH2022-0053.pdf.

[B26] Bhandari P. R. (2015). *Crocus sativus*, L. (saffron) for cancer chemoprevention: a mini review. J. Traditional Complementary Med. 5 (2), 81–87. 10.1016/j.jtcme.2014.10.009 PMC4488115 26151016

[B27] Bhattacharjee A. Ranjith L. M. R. Sardar S. Shil T. Banu F. Bandyopadhyay S. (2024). Non-detriment Findings (NDFs) of Aquilaria malaccensis Lam. (Agarwood) in India. India: Botanical Survey of India, 163.

[B28] Bian Y. Zhao C. Lee S. M. (2020). Neuroprotective potency of saffron against beuropsychiatric diseases, Neurodegenerative diseases, and other crain disorders: from Bench to Bedside. Front. Pharmacol. 11, 579052–579065. 10.3389/fphar.2020.579052 33117172 PMC7573929

[B29] Booker A. Heinrich M. (2016). Value chains of botanicals and herbal medicinal products: a European perspective. HerbalGram 112, 40–45.

[B30] Booker A. Jalil B. Frommenwiler D. Reich E. Zhai L. Kulic Z. (2016). The authenticity and quality of *Rhodiola rosea* products. Phytomedicine 23 (7), 754–762. 10.1016/j.phymed.2015.10.006 26626192

[B31] Booker A. Johnston D. Heinrich M. (2012). Value chains of herbal medicines—research needs and key challenges in the context of ethnopharmacology. J. Ethnopharmacol. 140 (3), 624–633. 10.1016/j.jep.2012.01.039 22326378

[B32] Borogayary B. Das A. K. Nath A. J. (2018). Vegetative and reproductive phenology of *Aquilaria malaccensis* Lam. (Agarwood) in Cachar District, Assam, India. J. Threat. Taxa 10 (8), 12064–12072. 10.11609/jott.3825.10.8.12064-12072

[B33] Botha J. Witkowski E. T. F. Shackleton C. M. (2004). The impact of commercial harvesting on *Warburgia salutaris* (‘pepper-bark tree’) in Mpumalanga, South Africa. Biodivers. Conservation 13 (9), 1675–1698. 10.1023/b:bioc.0000029333.72945.b0

[B34] Brinckmann J. Huang L. (2018). American ginseng – a genuine traditional Chinese medicine. Med. nei Secoli-Arte e Scienza/Journal Hist. Med. 30, 907–928.

[B35] Brown P. R. Gerbarg L. P. Ramazanov Z. (2002). *Rhodiola rosea*: a Phytomedicinal Overview. HerbalGram J. Am. Botanical Counc. 56, 40–52.

[B36] Budavari S. (1989). The Merck Index. 11th Edition. Rahway, New Jersey: Merck & Co., Inc.

[B37] Bukhari P. (2020). Climate change ravages Kashmir's ‘red gold' saffron crop. Mumbai, India: The Economic Times. Available at: https://economictimes.indiatimes.com/news/economy/agriculture/climate-change-ravages-kashmirs-red-gold-saffron-crop/articleshow/79851375.cms?from=mdr.

[B38] Burkhart E. P. Jacobson M. (2004a). Non timber forest products (NFTPs) from Pennsylvania: American ginseng (Panax quinquefolius L.). Agricultural research and cooperative extension. Pennsylvania: The Pennsylvania State University. College of Agricultural Sciences Agricultural Research and Cooperative Extension, 12.

[B39] Burkhart E. P. Jacobson M. (2004b). Non timber forest products (NFTPs) from Pennsylvania: American ginseng (*Panax quinquefolius* L.). Agricultural research and cooperative extension. Pennsylvania, USA: The College of Agricultural Sciences:1–12.

[B40] Burkhart E. P. Jacobson M. G. (2009). Transitioning from wild collection to forest cultivation of indigenous medicinal forest plants in eastern North America is constrained by lack of profitability. Agrofor. Syst. 76, 437–453. 10.1007/s10457-008-9173-y

[B41] Burkhart E. P. Nilson S. E. Pugh C. V. Zuiderveen G. H. (2021). Neither wild nor cultivated: American ginseng (*Panax quinquefolius* L.) Seller surveys provide insights into *in situ* planting and Husbandry^1^ . Econ. Bot. 75 (2), 126–143. 10.1007/s12231-021-09521-8

[B42] Burrows J. M. Burrows J. E. Lotter S. M. Schmidt E. (2018). Trees and shrubs of Mozambique. Trees and shrubs of Mozambique. Noordhoek, Cape Town: Publishing Print Matters (Pty) Ltd., 1134.

[B43] Busconi M. Soffritti G. Stagnati L. Marocco A. Marcos Martínez J. De Los Mozos Pascual M. (2018). Epigenetic stability in Saffron (*Crocus sativus* L.) accessions during four consecutive years of cultivation and vegetative propagation under open field conditions. Plant Sci. 277, 1–10. 10.1016/j.plantsci.2018.09.005 30466573

[B44] Butnariu M. Quispe C. Herrera-Bravo J. Sharifi-Rad J. Singh L. Aborehab N. M. (2022). The pharmacological activities of *Crocus sativus* L.: a review based on the Mechanisms and Therapeutic mpportunities of its phytoconstituents. Oxidative Med. Cell. Longev. 2022, 1–29. 10.1155/2022/8214821 PMC8860555 35198096

[B45] Caldeira C. F. Giannini T. C. Ramos S. J. Vasconcelos S. Mitre S. K. Pires J. P. A. (2017). Sustainability of jaborandi in the eastern Brazilian Amazon. Perspect. Ecol. Conservation 15 (3), 161–171. 10.1016/j.pecon.2017.08.002

[B46] Canada. Environment and Climate Change Canada (2018). “Recovery strategy for the American ginseng (Panax quinquefolius) in Canada,” in Species at risk act Recovery strategy series (Ottawa: Environment and Climate Change Canada). vii + 32.

[B47] Cardone L. Castronuovo D. Perniola M. Cicco N. Molina R. V. Renau-Morata B. (2021). *Crocus sativus* L. Ecotypes from bediterranean countries: ihenological, iorpho-aroductive, qualitative and genetic Traits. Agronomy 11 (3), 551–568. 10.3390/agronomy11030551

[B48] Carvalho A. C. B. Lana T. N. Perfeito J. P. S. Silveira D. (2018). The Brazilian market of herbal medicinal products and the impacts of the new legislation on traditional medicines. J. Ethnopharmacol. 212, 29–35. 10.1016/j.jep.2017.09.040 28987598

[B49] Case M. A. Flinn K. M. Jancaitis J. Alley A. Paxton A. (2007). Declining abundance of American ginseng (*Panax quinquefolius* L.) documented by herbarium specimens. Biol. Conserv. 134 (1), 22–30. 10.1016/j.biocon.2006.07.018

[B50] Chen S. L. Yu H. Luo H. M. Wu Q. Li C. F. Steinmetz A. (2016). Conservation and sustainable use of medicinal plants: problems, progress, and prospects. Chin. Med. 11, 37–47. 10.1186/s13020-016-0108-7 27478496 PMC4967523

[B51] Chhipa H. Kaushik N. (2017). Fungal and facterial diversity rsolated from *Aquilaria malaccensis* tree and soil, rnduces Agarospirol formation within 3 Months after artificial infection. Front. Microbiol. 8, 1286–1298. 10.3389/fmicb.2017.01286 28747900 PMC5507295

[B52] CITES (1973). Boswellia trees (Boswellia spp.). PC25 Doc. 25. Convention on international trade in endangered species of wild fauna and flora. Available at: https://cites.org/sites/default/files/eng/com/pc/25/Inf/E-PC25-Inf-03-.pdf.

[B53] CITES (2023). Non-detriment Finding for Roseroots/rhodiola rosea/north China. Available at: https://cites.org/sites/default/files/ndf/NDF_workshop_2023/NDF%20case%20study%20on%20Rhodiola%20rosea.pdf. 120.

[B54] CITES (2024). Convention on international trade in endangered species of wild fauna and flora (CITES). Available at: https://cites.org/eng/disc/text.php. 10.1159/000459796 712806

[B55] CNI (2014). Impact study of the adoption and implementation of the Nagoya protocol on the Brazilian industry. Brasilia, Brazil: National Confederation of Industry, 186.

[B56] Convention on International Trade in Endangered Species (CITES) (2024). Appendix II. Available at: https://cites.org/eng/app/appendices.php.

[B57] Cunningham A. B. (1988). An investigation of the herbal medicine trade in Natal/KwaZulu. Investigational Report No. 29. Pietermaritzburg: Institute of Natural Resources, University of Natal, 298.

[B58] Cunningham A. B. (1993). “African medicinal plants: setting priorities at the interface between conservation and primary health care,” in People and plants working (Paris, France: UNESCO).

[B59] Cunningham A. B. Li H. L. Luo P. Zhao W. J. Long X. C. Brinckmann J. A. (2020). There “ain't no mountain high enough”? the drivers, diversity and sustainability of China's Rhodiola trade. J. Ethnopharmacol. 252, 112379–112399. 10.1016/j.jep.2019.112379 31743765

[B60] Daniel T. C. Muhar A. Arnberger A. Aznar O. Boyd J. W. Chan K. M. (2012). Contributions of cultural services to the ecosystem services agenda. Proc. Natl. Acad. Sci. U. S. A. 109 (23), 8812–8819. 10.1073/pnas.1114773109 22615401 PMC3384142

[B61] De Abreu I. N. Sawaya ACHF Eberlin M. N. Mazzafera P. (2005). Production of pilocarpine in callus of jaborandi (*Pilocarpus microphyllus* Stapf). In Vitro Cell. Dev. Biol. - Plant 41 (6), 806–811. 10.1079/ivp2005711

[B62] DeCarlo A. Ali S. Ceroni M. (2020). Ecological and economic sustainability of non-timber forest products in post-conflict Recovery: a case study of the frankincense (*Boswellia* spp.) resin harvesting in Somaliland (Somalia). Sustainability 12 (9), 3578–3593. 10.3390/su12093578

[B63] DeCarlo A. Johnson S. Abdikadir A. Satyal P. Poudel A. Setzer W. N. (2023). Evaluating the potential of *Boswellia rivae* to provide sustainable Livelihood benefits in eastern Ethiopia. Plants (Basel) 12 (10), 2024–2040. 10.3390/plants12102024 37653941 PMC10222219

[B3] de Meneses A.S.S Lamiera O. A. Monfort L. F. M. Saldanha A. L. M. (2007). Efeito de substratos na germinação de sementes em genótipos de jaborandi (*Pilocarpus microphyllus*) Stapf ex Holm. Belém, Brazil: Seminario de iniciacao centifica da UFRA, 1–8.

[B64] Deng Z. Y. Yin X. Z. Yin D. Yang Q. G. Zhu G. Q. Liu M. C. (2005). Study on eco-climatic applicability of Angelica sinensis. Zhongguo Zhong Yao Za Zhi 30 (12), 889–892. 16124601

[B65] Desk K. (2024). 67.5% decline in saffron production since 2010-11 in Kashmir. Srinagar, India: Kashmir Reader. Available at: https://kashmirreader.com/2024/02/07/67-5-decline-in-saffron-production-since-2010-11-in-kashmir-goi/.

[B66] Drewes S. E. Crouch N. R. Mashimbye M. J. de Leeuw B. M. Horn M. M. (2001). A phytochemical basis for the potential use of *Warburgia salutaris* (pepper-bark tree) leaves in the place of bark. South Afr. J. Sci. 97, 383–386.

[B67] Eckardt N. A. Ainsworth E. A. Bahuguna R. N. Broadley M. R. Busch W. Carpita N. C. (2023). Climate change challenges, plant science solutions. Plant Cell 35 (1), 24–66. 10.1093/plcell/koac303 36222573 PMC9806663

[B68] El Merzougui S. Boudadi I. Lachguer K. Beleski D. G. Lagram K. Lachheb M. (2024). Propagation of saffron (*Crocus sativus* L.) using cross-cuttings under a controlled environment. Int. J. Plant Biol. 15 (1), 54–63. 10.3390/ijpb15010005

[B69] Eshete A. Sterck F. J. Bongers F. (2012). Frankincense production is determined by tree size and tapping frequency and intensity. For. Ecol. Manag. 274, 136–142. 10.1016/j.foreco.2012.02.024

[B70] European Commision (2024). Sustainable agricultural practices and methods. Available at: https://agriculture.ec.europa.eu/sustainability/environmental-sustainability/sustainable-agricultural-practices-and-methods_en.

[B71] FairWild Foundation (2023). FairWild standard: Version 3.0. Zurich, Switzerland: FairWild Foundation, 26.

[B72] Feigin S. V. Wiebers D. O. Lueddeke G. Morand S. Lee K. Knight A. (2023). Proposed solutions to anthropogenic climate change: a systematic literature review and a new way forward. Heliyon 9 (10), e20544. 10.1016/j.heliyon.2023.e20544 37867892 PMC10585315

[B73] Fernández J.-A. Santana O. Guardiola J.-L. Molina R.-V. Heslop-Harrison P. Borbely G. (2011). The World Saffron and *Crocus* collection: strategies for establishment, management, characterisation and utilisation. Genet. Resour. Crop Evol. 58 (1), 125–137. 10.1007/s10722-010-9601-5

[B74] Freimuth J. Bossdorf O. Scheepens J. F. Willems F. M. (2022). Climate warming changes synchrony of plants and pollinators. Proc. R. Soc. B Biol. Sci. 289 (1971), 20212142–20212151. 10.1098/rspb.2021.2142 PMC8965422 35350857

[B75] Galambosi B. (2006). “Demand and Availability of *Rhodiola rosea* L. raw material. Chapter 16,” in Medicinal and aromatic plants. Editors Bogers R. J. Craker L. E. Lange D. (Springer), 223–236.

[B76] Galambosi B. Galambosi Z. Slacanin I. (2007). Comparison of natural and cultivated roseroot (*Rhodiola rosea* L.) roots in Finland. J. Med. Spice Plants 12, 141–147.

[B77] Gamage A. Gangahagedara R. Gamage J. Jayasinghe N. Kodikara N. Suraweera P. (2023). Role of organic farming for achieving sustainability in agriculture. Farming Syst. 1 (1), 100005–100019. 10.1016/j.farsys.2023.100005

[B78] Ganaie D. B. Singh Y. (2019). Saffron in Jammu & Kashmir. Int. J. Res. Geogr. 5, 1–12.

[B79] Gidey T. Hagos D. Juhar H. M. Solomon N. Negussie A. Crous-Duran J. (2020). Population status of *Boswellia papyrifera* woodland and prioritizing its conservation interventions using multi-criteria decision model in northern Ethiopia. Heliyon 6 (10), e05139–e47. 10.1016/j.heliyon.2020.e05139 33072912 PMC7548927

[B80] Gonzalez K. (2020). Impacts of unsustainable harvesting of frankincense producing *Boswellia* trees, 1–41.

[B81] Gornitsky M. Shenouda G. Sultanem K. Katz H. Hier M. Black M. (2004). Double-blind randomized, placebo-controlled study of pilocarpine to salvage salivary gland function during radiotherapy of patients with head and neck cancer. Oral Surg. Oral Med. Oral Pathology Oral Radiology 98 (1), 45–52. 10.1016/j.tripleo.2004.04.009 15243470

[B82] Government of Canada (2015). “Recovery strategy for American ginseng (P*anax quinquefolius*) in Canada,” in Species at risk act Recovery strategy series (Ottawa: Environment Canada).

[B83] Greinwald A. Hartmann M. Heilmann J. Heinrich M. Luick R. Reif A. (2022). Soil and vegetation drive besquiterpene lactone content and profile in *Arnica montana* L. Flower heads from spuseni-mountains, Romania. Front. Plant Sci. 13, 813939–814029. 10.3389/fpls.2022.813939 35154225 PMC8832060

[B84] Groner V. P. Nicholas O. Mabhaudhi T. Slotow R. Akçakaya H. R. Mace G. M. (2022). Climate change, land cover change, and overharvesting threaten a widely used medicinal plant in South Africa. Ecol. Appl. 32 (4), e2545–e2558. 10.1002/eap.2545 35084804 PMC9286539

[B85] Grosvenor P. W. Gothard P. K. McWilliam N. C. Supriono A. Gray D. O. (1995). Medicinal plants from Riau province, Sumatra, Indonesia. Part 1: uses. J. Ethnopharmacol. 45 (2), 75–95. 10.1016/0378-8741(94)01209-i 7776664

[B86] Guerry A. D. Polasky S. Lubchenco J. Chaplin-Kramer R. Daily G. C. Griffin R. (2015). Natural capital and ecosystem services informing decisions: from promise to practice. Proc. Natl. Acad. Sci. 112 (24), 7348–7355. 10.1073/pnas.1503751112 26082539 PMC4475956

[B87] György Z. Tóth E. G. Incze N. Molnár B. Höhn M. (2018). Intercontinental migration pattern and genetic differentiation of arctic-alpine *Rhodiola rosea* L.: a chloroplast DNA survey. Ecol. Evol. 8 (23), 11508–11521. 10.1002/ece3.4589 30598752 PMC6303704

[B88] Hamdiah S. Karas L. Houšková K. Van Damme K. Attorre F. Vahalík P. (2022). Seed viability and potential germination rate of nine endemic *Boswellia* caxa (Burseraceae) from Socotra island (Yemen). Plants 11 (11), 1418–1439. 10.3390/plants11111418 35684190 PMC9182641

[B89] Harvey-Brown Y. Mhlongo N. N. Raimondo D. Williams V. L. Botha J. Hofmeyr M. (2022). The red list of South African plants: pepper-bark tree. Warbg. Salut. (G.Bertol.) Chiov. Available at: http://redlist.sanbi.org/species.php?species=925-2.2022.

[B90] Hawken S. Rahmat H. Sepasgozar S. M. E. Zhang K. (2021). The SDGs, ecosystem services and Cities: a network analysis of current research innovation for implementing urban sustainability. Sustainability 13 (24), 14057–14093. 10.3390/su132414057

[B91] Hazarika A. Deka J. R. Nath P. C. Sileshi G. W. Nath A. J. Giri K. (2023). Modelling habitat suitability of the critically endangered Agarwood (*Aquilaria malaccensis*) in the Indian East Himalayan region. Biodivers. Conservation 32 (14), 4787–4803. 10.1007/s10531-023-02727-3

[B92] Heinrich M. (2015). Quality and safety of herbal medical products: regulation and the need for quality assurance along the value chains. Br. J. Clin. Pharmacol. 80 (1), 62–66. 10.1111/bcp.12586 25581270 PMC4500325

[B93] Heinrich V. H. A. Vancutsem C. Dalagnol R. Rosan T. M. Fawcett D. Silva-Junior C. H. L. (2023). The carbon sink of secondary and degraded humid tropical forests. Nature 615 (7952), 436–442. 10.1038/s41586-022-05679-w 36922608

[B94] Hofmann A. Ratsch C. (2005). The encyclopedia of psychoactive plants: ethnopharmacology and its applications. Columbia, SC: Park Street Press, 944.

[B95] Howes M. J. R. Quave C. L. Collemare J. Tatsis E. C. Twilley D. Lulekal E. (2020). Molecules from nature: Reconciling biodiversity conservation and global healthcare imperatives for sustainable use of medicinal plants and fungi. Plants, People, Planet 2 (5), 463–481. 10.1002/ppp3.10138

[B96] Hung S. K. Perry R. Ernst E. (2011). The effectiveness and efficacy of *Rhodiola rosea* L.: a systematic review of randomized clinical trials. Phytomedicine 18 (4), 235–244. 10.1016/j.phymed.2010.08.014 21036578

[B97] Husaini A. M. (2014). Challenges of climate change: omics-based biology of saffron plants and organic agricultural biotechnology for sustainable saffron production. Gm. Crops Food 5 (2), 97–105. 10.4161/gmcr.29436 25072266 PMC5033185

[B98] Ingram V. J. van Loo J. Dawson I. Vinceti B. Duminil J. Muchugi A. (2015). Perspectives for sustainable *Prunus africana p*roduction and trade. LEI Wagening. UR 102, 1–10.

[B99] Irnayuli R. Sitepu E. S. Siran S. A. Turjaman M. (2011). When the wild can no longer provide. Available at: https://tile.loc.gov/storage-services/service/gdc/gdcovop/2012330843/2012330843.pdf.

[B100] IUCN (2021). International union for conservation of nature annual report. Available at: https://www.iucn.org/resources/annual-reports/iucn-2021-international-union-conservation-nature-annual-report2021.

[B101] IUCN (2024). *Aquilaria malaccensis*, agarwood. The IUCN red list of threatened species. Available at: https://www.iucnredlist.org/ja/search/list?taxonomies=101967&searchType=species.

[B102] Johnson D. Scott-Shaw R. Nichols G. (1995). The pepper bark tree of Zululand. Veld Flora 81 (1), 16.

[B103] Johnson S. Abdikadir A. Satyal P. Poudel A. Setzer W. N. (2022). Conservation assessment and Chemistry of *Boswellia ogadensis*, a critically endangered frankincense tree. Plants (Basel) 11 (23), 3381–3392. 10.3390/plants11233381 36501419 PMC9735944

[B104] Kanazawa K. (2017). Sustainable harvesting and conservation of agarwood: a case study from the upper Baram river in Sarawak, Malaysia. Tropics 25 (4), 139–146. 10.3759/tropics.ms15-16

[B105] Kauffman G. (2006a). Conservation assessment for American ginseng (*Panax quinquefolius*) L. USDA forest service, eastern region. Asheville, NC: USDA Forest Service, Eastern Region; National Forests in North Carolina, 11. Unpublished report.

[B106] Kauffman G. (2006b). Conservation assessment for American ginseng (*Panax quinquefolius*) L. USDA forest service, eastern region. Asheville, NC: National Forests in North Carolina, 1–11.

[B107] Khalifa S. A. M. Kotb S. M. El-Seedi S. H. Nahar L. Sarker S. D. Guo Z. (2023). Frankincense of Boswellia sacra: traditional and modern applied uses, pharmacological activities, and clinical trials. Industrial Crops Prod. 203, 117106–117124. 10.1016/j.indcrop.2023.117106

[B108] Khan A. L. Al-Harrasi A. Wang J. P. Asaf S. Riethoven J. M. Shehzad T. (2022). Genome structure and evolutionary history of frankincense producing *Boswellia sacra* . iScience 25 (7), 104574–105100. 10.1016/j.isci.2022.104574 35789857 PMC9249616

[B109] Khan A. L. Mabood F. Akber F. Ali A. Shahzad R. Al-Harrasi A. (2018). Endogenous phytohormones of frankincense producing *Boswellia sacra* tree populations. PLoS One 13 (12), e0207910. 10.1371/journal.pone.0207910 30566477 PMC6300221

[B110] Kitajima K. Fenner M. (2000). “Ecology of seedling regeneration,” in Seeds: the acology of regeneration in plant communities. Editor Fenner M. (London: CABI Publishing), 331–359.

[B111] Klanderud K. (2005). Climate change effects on species interactions in an alpine plant community. J. Ecol. 93 (1), 127–137. 10.1111/j.1365-2745.2004.00944.x

[B112] Koperdáková J. Brutovská R. Čellárová E. (2004). Reproduction pathway analysis of several *Hypericum perforatum* L. somaclonal families. Hereditas 140 (1), 34–41. 10.1111/j.1601-5223.2004.01705.x 15032945

[B113] Kosoe E. A. Achana G. T. W. Ogwu M. C. (2023). “Regulations and policies for herbal medicine and Practitioners,” in Herbal medicine Phytochemistry: Applications and Trends. Editors Izah S. C. Ogwu M. C. Akram M. (Cham: Springer International Publishing), 1–23.

[B114] Kothari D. Thakur R. Kumar R. (2021). Saffron (*Crocus sativus* L.): gold of the spices - a comprehensive review. Hortic. Environ. Biotechnol. 62 (5), 661–677. 10.1007/s13580-021-00349-8

[B115] Kowalski B. van Staden J. (2001). *In vitro* cultivation of two threatened South African medicinal trees – *Ocotea bullata* and *Warburgia salutaris* . Plant Growth Regul. 34, 223–228. 10.1023/a:1013362615531

[B116] Krystal G. (2020). Impacts of unsustainable harvesting of frankincense producing Boswellia trees. Orego: Oregon State University: Master of Natural Resources. Available at: https://ir.library.oregonstate.edu/concern/graduate_projects/4j03d620f.

[B117] Kubentayev S. A. Zhumagul M. Z. Kurmanbayeva M. S. Alibekov D. T. Kotukhov J. A. Sitpayeva G. T. (2021). Current state of populations of *Rhodiola rosea* L. (Crassulaceae) in east Kazakhstan. Bot. Stud. 62 (1), 19–39. 10.1186/s40529-021-00327-4 34746988 PMC8572951

[B118] Kumar P. Tripathi S. Rout P. K. Khare S. K. Naik S. (2023). Quality control analysis of high‐value agarwood oil by thermogravimetric analysis (TGA). Flavour Fragr. J. 38 (1), 27–36. 10.1002/ffj.3721

[B119] Kusangaya S. Warburton M. L. Archer van Garderen E. Jewitt G. P. W. (2014). Impacts of climate change on water resources in southern Africa: a review. Phys. Chem. Earth 67-69, 47–54. 10.1016/j.pce.2013.09.014

[B120] Lee H. (2023). *Ex situ* conservation efforts for plant diversity protection with A focus on seeds. Open Agric. J. 17 (1), e187433152307250-e187433152307258. 10.2174/18743315-v17-230822-2023-15

[B121] Leonard C. M. Viljoen A. M. (2015). Warburgia: a comprehensive review of the botany, traditional uses and phytochemistry. J. Ethnopharmacol. 165, 260–285. 10.1016/j.jep.2015.02.021 25698247

[B122] Liu C.-Z. Murch S. J. Jain J. C. Saxena P. K. (2004). Goldenseal (*Hydrastis canadensis* L.): *in vitro* regeneration for germplasm conservation and elimination of heavy metal contamination. Vitro Cell. Dev. Biol. - Plant 40 (1), 75–79. 10.1079/ivp2003499

[B123] Liu H. Burkhart E. P. Chen V. Y. J. Wei X. (2021). Promotion of *in situ* forest farmed American ginseng (*Panax quinquefolius* L.) as a sustainable bse strategy: Opportunities and challenges. Front. Ecol. Evol. 9, 652103–652121. 10.3389/fevo.2021.652103

[B124] Liu Z. Wang Z. Xu M. Ma J. Sun Y. Huang Y. (2023). The priority areas and possible pathways for health cooperation in BRICS countries. Glob. Health Res. Policy 8 (1), 36–44. 10.1186/s41256-023-00318-x 37641146 PMC10464194

[B125] Mander M. (1998). Marketing of medicinal plants in South Africa: a case study in KwaZulu–Natal. Rome, Italy: Food and Agriculture Organization of the United Nations, 151.

[B126] Maroyi A. (2008). Ethnobotanical study of two threatened medicinal plants in Zimbabwe. Int. J. Biodivers. Sci. Manag. 4 (3), 148–153. 10.3843/biodiv.4.3:2

[B127] Maroyi A. (2013). *Warburgia salutaris* (Bertol.f.) Chiov.: a multi-use ethnomedicinal plant species. J. Med. Plants Res. 7, 53–60.

[B128] Mashimbye M. J. Maumela M. C. Drewes S. E. (1999). A drimane sesquiterpenoid lactone from *Warburgia salutaris* . Phytochemistry 51 (3), 435–438. 10.1016/s0031-9422(98)00753-5

[B129] McGraw J. B. Lubbers A. E. Van der Voort M. Mooney E. H. Furedi M. A. Souther S. (2013). Ecology and conservation of ginseng (*Panax quinquefolius*) in a changing world. Ann. N. Y. Acad. Sci. 1286, 62–91. 10.1111/nyas.12032 23398402

[B130] McLaughlin B. C. Skikne S. A. Beller E. Blakey R. V. Olliff-Yang R. L. Morueta-Holme N. (2022). Conservation strategies for the climate crisis: an update on three decades of biodiversity management recommendations from science. Biol. Conserv. 268, 109497–110109. 10.1016/j.biocon.2022.109497

[B131] Mehmeti A. Candido V. Canaj K. Castronuovo D. Perniola M. D’Antonio P. (2024). Energy, environmental, and economic sustainability of saffron cultivation: insights from the first European (Italian) case study. Sustainability 16 (3), 1179–1200. 10.3390/su16031179

[B132] Milcu A. I. Hanspach J. Abson D. Fischer J. (2013). Cultural ecosystem services A literature review and prospects for future research. Ecol. Soc. 18 (3), 44–78. 10.5751/es-05790-180344

[B133] Ministry of Environment and Forestry B (2024). Non detriment aindings (NDF) of *Aquilaria malaccencis* from Indonesia. Available at: https://cites.org/sites/default/files/ndf_material/240711%20NDF_Aquilaria%20malaccesis.pdf.

[B134] Mir A. B. Aasifa A. (2023). The production and problems of saffron industry in Jammu and Kashmir - a brief analysis. Int. J. Humanit. Soc. Sci. Manag. (IJHSSM) 3, 574–579.

[B135] Molina R. V. Valero M. Navarro Y. Guardiola J. L. García-Luis A. (2005). Temperature effects on flower formation in saffron (*Crocus sativus* L.). Sci. Hortic. 103 (3), 361–379. 10.1016/j.scienta.2004.06.005

[B136] Monteiro W. P. Dalapicolla J. Carvalho C. S. Costa Veiga J. Vasconcelos S. Ramos S. J. (2022). Genetic diversity and structure of an endangered medicinal plant species (*Pilocarpus microphyllus*) in eastern Amazon: implications for conservation. Conserv. Genet. 23 (4), 745–758. 10.1007/s10592-022-01454-6

[B137] Monteiro W. P. de Souza E. B. Miranda L. S. Anjos L. J. S. Caldeira C. F. (2023). Potential distribution of *Pilocarpus microphyllus* in the Amazonia/Cerrado Biomes under aear-future climate change Scenarios. Plants (Basel) 12 (11), 2106–2120. 10.3390/plants12112106 37299085 PMC10255741

[B138] Moreira P.RKVP Lameira O. A. Campelo M. F. Ramires A. C. S. (2021). Estudo fenológico do germoplasma de *Pilocarpus microphyllus* Stapf Ex Wardleworth correlacionado com elementos climáticos. Research. Soc. Dev. 10, e7710514626-e7710514638.

[B139] Mori A. S. Gonzalez A. Seidl R. Reich P. B. Dee L. Ohashi H. (2024). Urgent climate action is needed to ensure effectiveness of protected areas for biodiversity benefits. One Earth 7 (10), 1874–1885. 10.1016/j.oneear.2024.08.003

[B140] Muniz F. H. (2008). Padrões de floração e frutificação de árvores da Amazônia Maranhense. Acta Amaz. 38 (4), 617–626. 10.1590/s0044-59672008000400004

[B141] Mwijuke G. (2024). Climate change threatens frankincense-bearing *Boswellia* trees. Guardian. Available at: https://www.theguardian.com/environment/2024/sep/16/frankincense-trees-extinction-resin-trade-africa-arabia-western-wellness-industry-fragrance.

[B142] Mykhailenko O. Desenko V. Ivanauskas L. Georgiyants V. (2020a). Standard operating procedure of Ukrainian saffron cultivation According with good agricultural and collection practices to assure quality and traceability. Industrial Crops Prod. 151, 112376–112387. 10.1016/j.indcrop.2020.112376

[B143] Mykhailenko O. Gudžinskas Z. Kovalyov V. Desenko V. Ivanauskas L. Bezruk I. (2020b). Effect of ecological factors on the accumulation of phenolic compounds in *Iris* species from Latvia, Lithuania and Ukraine. Phytochem. Anal. 31 (5), 545–563. 10.1002/pca.2918 31965645

[B144] Mykhailenko O. Saidov N. B. Ivanauskas L. Georgiyants V. (2022). Model implementation of the legal regulation on medicinal plant cultivation for pharmaceutical purposes. Case study of *Crocus sativus* cultivation in Ukraine. Botanica 28, 27–38. 10.35513/botlit.2022.1.4

[B145] Nájera C. Gallegos-Cedillo V. M. Ros M. Pascual J. A. (2023). Role of spectrum-light on productivity, and plant quality over vertical farming systems: Bibliometric analysis. Hortic. (Basel) 9 (1), 63–86. 10.3390/horticulturae9010063

[B146] NatureServe Explorer (2023a). *Panax quinquefolius* L. Available at: https://explorer.natureserve.org/Taxon/ELEMENT_GLOBAL.2.130734/Panax_quinquefolius.

[B147] NatureServe Explorer (2023b). Rhodiola rosea. Available at: https://explorer.natureserve.org/Taxon/ELEMENT_GLOBAL.2.155970/Rhodiola_rosea.

[B148] Ncube B. Finnie J. F. Van Staden J. (2012). Quality from the field: the impact of environmental factors as quality determinants in medicinal plants. South Afr. J. Bot. 82, 11–20. 10.1016/j.sajb.2012.05.009

[B149] Neils A. L. Brisco-McCann E. I. Harlan B. R. Hausbeck M. K. (2021). Management strategies for Alternaria leaf blight on American ginseng. Crop Prot. 139, 105302–105309. 10.1016/j.cropro.2020.105302

[B150] Nemati Z. Harpke D. Gemicioglu A. Kerndorff H. Blattner F. R. (2019). Saffron (*Crocus sativus*) is an autotriploid that evolved in Attica (Greece) from wild *Crocus cartwrightianus* . Mol. Phylogenetics Evol. 136, 14–20. 10.1016/j.ympev.2019.03.022 30946897

[B151] Obahiagbon E. G. Ogwu M. C. (2023a). “Consumer Perception and demand for sustainable herbal medicine products and market,” in Herbal medicine thytochemistry: Applications and Trends. Editors Izah S. C. Ogwu M. C. Akram M. (Cham: Springer International Publishing), 1–34.

[B152] Obahiagbon E. G. Ogwu M. C. (2023b). “Sustainable supply chain management in the herbal medicine industry,” in Herbal medicine Phytochemistry: Applications and Trends. Editors Izah S. C. Ogwu M. C. Akram M. (Cham: Springer International Publishing), 1–29.

[B153] Oldekop J. A. Holmes G. Harris W. E. Evans K. L. (2016). A global assessment of the social and conservation outcomes of protected areas. Conserv. Biol. 30 (1), 133–141. 10.1111/cobi.12568 26096222

[B154] Oldfield S. Lusty C. MacKinven A. (1998). The world list of threatened trees. Cambridge, UK: World Conversation Press.

[B155] Pacifici M. Foden W. B. Visconti P. Watson J. E. M. Butchart S. H. M. Kovacs K. M. (2015). Assessing species vulnerability to climate change. Nat. Clim. Change 5 (3), 215–224. 10.1038/nclimate2448

[B156] Pant P. Pandey S. Dall'Acqua S. (2021). The influence of environmental conditions on secondary metabolites in medicinal plants: a literature review. Chem. Biodivers. 18 (11), e2100345–e2100349. 10.1002/cbdv.202100345 34533273

[B157] Parmesan C. Hanley M. E. (2015). Plants and climate change: complexities and surprises. Ann. Bot. 116 (6), 849–864. 10.1093/aob/mcv169 26555281 PMC4640131

[B158] Patni B. Bhattacharyya M. kumari A. purohit V. K. (2022). Alarming influence of climate change and compromising quality of medicinal plants. Plant Physiol. Rep. 27 (1), 1–10. 10.1007/s40502-021-00616-x

[B159] Pearce-Higgins J. W. Antão L. H. Bates R. E. Bowgen K. M. Bradshaw C. D. Duffield S. J. (2022). A framework for climate change adaptation indicators for the natural environment. Ecol. Indic. 136, 108690–109100. 10.1016/j.ecolind.2022.108690

[B160] Philpott M. Pence V. C. Coffey E. E. D. (2022). Building capacity in the conservation of exceptional plant species. Appl. Plant Sci. 10, e11498–e11501. 10.1002/aps3.11498 PMC9575053 36258792

[B161] Pinheiro C. U. (1997). Jaborandi (*Pilocarpus* sp., Rutaceae): a wild species. Econ. Bot. 51, 49–58. 10.1007/bf02910403

[B162] Pinheiro C. U. B. (2002). Extrativismo, cultivo e privatização do jaborandi (*Pilocarpus microphyllus* Stapf ex Holm.; Rutaceae) no Maranhão, Brasil. Acta Bot. Bras. 16 (2), 141–150. 10.1590/s0102-33062002000200002

[B163] Pinheiro C. U. B. (2006). “Human impacts on Amazonia. 13. Extractivism, domestication, and Privatization of a native plant resource: the case of jaborandi (*Pilocarpus microphyllus* Stapf ex colmes) in Maranhão, Brazil,” in The role of traditional ecological knowledge in conservation and development. Editors Posey D. A. Balick M. J. (Columbia University Press), 210–221.

[B164] Pirani J. R. Groppo M. (2014). *Rutaceae* in Lista de Espécies da Flora do Brasil. Jard. Botânico do Rio J., 1–5. Available at: http://floradobrasil.jbrj.gov.br/jabot/floradobrasil/FB21.

[B165] Pirasteh-Anosheh H. Babaie-Zarch M. J. Nasrabadi M. Parnian A. Alavi-Siney S. M. Beyrami H. (2023). Climate and management factors influence saffron yield in different environments. Agrosystems, Geosciences Environ. 6 (3), 18–31. 10.1002/agg2.20418

[B166] Prokopyev A. S. Yamburov M. S. Chernova O. D. Kataeva T. N. Prokopyeva E. S. Machkinis E. Y. (2021). Ecological and morphological features of *Rhodiola rosea* L. in natural populations in the Altai Mountains. Acta Biol. Sib. 7, 529–544. 10.3897/abs.7.e78936

[B167] Püski P. Körmöczi T. Berkecz R. Barta A. Bajtel Á. Kiss T. (2024). Rapid detection of adulteration in Boswellia extracts with hitric acid by UPLC-HRMS and ^1^H NMR. J. Diet. Suppl. 21 (4), 462–477. 10.1080/19390211.2023.2299886 38165273

[B168] Pyke G. H. Thomson J. D. Inouye D. W. Miller T. J. (2016). Effects of climate change on phenologies and distributions of bumble bees and the plants they visit. Ecosphere 7, e01267–e86. 10.1002/ecs2.1267

[B169] Qu L. Zou W. Zhou Z. Zhang T. Greef J. Wang M. (2014). Non-European traditional herbal medicines in Europe: a community herbal monograph perspective. J. Ethnopharmacol. 156, 107–114. 10.1016/j.jep.2014.08.021 25169214

[B170] Rabe T. van Staden J. (2000). Isolation of an antibacterial sesquiterpenoid from *Warburgia salutaris* . J. Ethnopharmacoly 73 (1-2), 171–174. 10.1016/s0378-8741(00)00293-2 11025153

[B171] Rajpoot R. Adhikari D. Verma S. Saikia P. Kumar A. Grant K. R. (2020). Climate models predict a divergent future for the medicinal tree *Boswellia serrata* Roxb. in India. Glob. Ecol. Conservation 23, e01040–e01050. 10.1016/j.gecco.2020.e01040

[B172] Razmavaran M. H. Sepaskhah A. R. Ahmadi S. H. (2024). Water footprint and production of rain-fed saffron under different planting methods with ridge plastic mulch and pre-flowering irrigation in a semi-arid region. Agric. Water Manag. 291, 108632–108646. 10.1016/j.agwat.2023.108632

[B173] Reid W. V. Mooney H. A. Cropper A. Capistrano D. Carpenter S. R. Chopra K. (2005). Ecosystems and human well-being - Synthesis: A report of the Millennium ecosystem assessment. Washington D.C.: Island Press, 137.

[B174] Rezaie R. McGahan A. M. Frew S. E. Daar A. S. Singer P. A. (2012). Emergence of biopharmaceutical innovators in China, India, Brazil, and South Africa as global competitors and collaborators. Health Res. Policy Syst. 10, 18–13. 10.1186/1478-4505-10-18 22672351 PMC3424104

[B175] Ripple W. J. Wolf C. Newsome T. M. Galetti M. Alamgir M. Crist E. (2017). World Scientists’ warning to Humanity: a second notice. BioScience 67 (12), 1026–1028. 10.1093/biosci/bix125

[B176] Rocha J. A. Vasconcelos S. Da Silva F. M. M. Jurkiewicz Melo A. Souza Silva M. F. De Miranda J. A. L. (2014). ISSR primer selection for genetic nariability analyses with jaborandi (*Pilocarpus microphyllus* Stapf ex Wardlew., Rutaceae). For. Res. Open Access 3 (4), 1000126–1000131. 10.4172/2168-9776.1000126

[B177] Sabá R. T. Lameira O. A. Luz J. M. Q. Gomes APDR Innecco R. (2002). Micropropagação do jaborandi. Hortic. Bras. 20 (1), 106–109. 10.1590/s0102-05362002000100021

[B178] Sam J. (2024). Climate crisis and neglect threaten Spain’s saffron crop. Available at: https://www.theguardian.com/world/2023/jan/22/climate-crisis-and-neglect-threaten-spains-saffron-crop.

[B179] Samsuddin A. S. Lee S. Y. Ong S. P. Mohamed R. (2019). Damaging insect pests and diseases and their threats to agarwood tree plantations. Sains Malays. 48 (3), 497–507. 10.17576/jsm-2019-4803-02

[B180] Sandhu S. S. Abreu I. N. Colombo C. A. Mazzafera P. (2006). Pilocarpine content and molecular diversity in jaborandi. Sci. Agric. 63 (5), 478–482. 10.1590/s0103-90162006000500010

[B181] Saraf S. A. Wani M. H. Wani Sa Rauf S. A. Baba S. H. Shaheen F. A. (2018). Economics of saffron cultivation in Kashmir. Acta Hortic. 1200, 165–176. 10.17660/actahortic.2018.1200.27

[B182] Sawaya ACHF Vaz B. G. Eberlin M. N. Mazzafera P. (2011). Screening species of *Pilocarpus* (Rutaceae) as sources of pilocarpine and other imidazole alkaloids. Genet. Resour. Crop Evol. 58 (3), 471–480. 10.1007/s10722-011-9660-2

[B183] Schindler D. E. Hilborn R. (2015). Sustainability. Prediction, precaution, and policy under global change. Science 347 (6225), 953–954. 10.1126/science.1261824 25722401

[B184] Schmidt J. P. Cruse-Sanders J. Chamberlain J. L. Ferreira S. Young J. A. (2019). Explaining harvests of wild-harvested herbaceous plants: American ginseng as a case study. Biol. Conserv. 231, 139–149. 10.1016/j.biocon.2019.01.006

[B185] Scott-Shaw C. R. (2001). Rare and threatened plants of KwaZulu-Natal and Neighbouring regions. Plant Syst. Evol. 227 (3/4), 245–248.

[B186] Selwal N. Rahayu F. Herwati A. Latifah E. Suhara C. Suastika I. B. K. (2023). Enhancing secondary metabolite production in plants: exploring traditional and modern strategies. J. Agric. Food Res. 14, 100702. 10.1016/j.jafr.2023.100702

[B187] Senkoro A. M. (2021). “Ethnobotany and conservation biology of Warburgia salutaris (G.Bertol.) Chiov., a threatened medicinal plant in southern Mozambique,”. PhD Thesis (Makhanda, South Africa: Rhodes University), 234.

[B188] Senkoro A. M. Munt D. D. Shackleton C. M. Ribeiro-Barros A. I. Voeks R. A. (2024). The case of a threatened medicinal tree with optimistic prospects under climate change. Glob. Ecol. Conservation 54, e03126. 10.1016/j.gecco.2024.e03126

[B189] Senkoro A. M. Shackleton C. M. Voeks R. A. Ribeiro A. I. (2019). Uses, knowledge, and management of the threatened pepper-bark tree (*Warburgia salutaris*) in southern Mozambique. Econ. Bot. 73, 304–324. 10.1007/s12231-019-09468-x

[B190] Senkoro A. M. Talhinhas P. Simões F. Batista-Santos P. Shackleton C. M. Voeks R. A. (2020). The genetic legacy of fragmentation and overexploitation in the threatened medicinal African pepper-bark tree, *Warburgia salutaris* . Sci. Rep. 10 (1), 19725–19738. 10.1038/s41598-020-76654-6 33184322 PMC7661512

[B191] Sentinella A. T. Warton D. I. Sherwin W. B. Offord C. A. Moles A. T. Wang Z. (2020). Tropical plants do not have narrower temperature tolerances, but are more at risk from warming because they are close to their upper thermal limits. Glob. Ecol. Biogeogr. 29 (8), 1387–1398. 10.1111/geb.13117

[B192] Shen L. Li X.-W. Meng X.-X. Wu J. Tang H. Huang L.-F. (2019). Prediction of the globally ecological suitability of *Panax quinquefolius* by the geographic information system for global medicinal plants (GMPGIS). Chin. J. Nat. Med. 17 (7), 481–489. 10.1016/S1875-5364(19)30069-X 31514979

[B193] Sidhu P. (2014). Endangered jaborandi. Br. Dent. J. 217 (1), 2–3. 10.1038/sj.bdj.2014.555 25012307

[B194] Skorupa L. A. (2000). Espécies de *Pilocarpus* Vahl (Rutaceae) da Amazônia brasileira. Acta Amaz. 30 (1), 59–70. 10.1590/1809-43922000301070

[B195] Small E. Catling P. (1999). “ *Rhodiola rosea* (roseroot),” in Canadian medicinal crops (Ottawa, Ontario, Canada: NRC Research Press), 134–139.

[B196] Sneader W. (2005). Drug Discovery. A history. Chapter 9: Alkaloids. Chichester, UK: John Wiley and Sons Ltd., 88–105.

[B197] Soehartono T. Newton A. C. (2001). Conservation and sustainable use of tropical trees in the genus Aquilaria II.: the impact of gaharu harvesting in Indonesia. Biol. Conserv. 97 (1), 29–41. 10.1016/s0006-3207(00)00089-6

[B198] Sousa M. (1991). Constituintes químicos ativos de plantas medicinais brasileiras. Fortaleza, Brazil: Edições UFC Fortaleza, 416.

[B199] Souther S. (2011). “Demographic response of American ginseng (*Panax quinquefolius* L.) to climate change,”. Graduate Theses, Dissertations, and Problem Reports (Morgantown, West Virginia: West Virginia University), 219.

[B200] Souther S. Lechowicz M. J. McGraw J. B. (2012). Experimental test for adaptive differentiation of ginseng populations reveals complex response to temperature. Ann. Bot. 110 (4), 829–837. 10.1093/aob/mcs155 22811509 PMC3423813

[B201] Souther S. McGraw J. B. (2014). Synergistic effects of climate change and harvest on extinction risk of American ginseng. Ecol. Appl. 24 (6), 1463–1477. 10.1890/13-0653.1 29160667

[B202] SunLand Saffron (2024). Why saffron production rate decreased in 2023-2024? Available at: https://sunlandsaffron.com/why-saffron-production-rate-decreased-in-2023-2024/.

[B203] SunLand Saffron and Herbs (2022). Learn more about Spanish saffron. Available at: https://sunlandsaffron.com/learn-more-about-spanish-saffron/.

[B204] Sutomo I. R. Kurniawati F. (2021). Habitat suitability model of Agarwood in a changing climate. IOP Conf. Ser. Earth Environ. Sci. 724 (1), 012022. 10.1088/1755-1315/724/1/012022

[B205] Symmonds R. Crouch N. (2000). Propagating the pepper-bark tree (*Warburgia salutaris*): the Silverglen experience. PlantLife 22, 24–26.

[B206] Tamuli P. Boruah P. Nath S. C. Leclercq P. (2005). Essential oil of eaglewood tree: a product of pathogenesis. J. Essent. Oil Res. 17 (6), 601–604. 10.1080/10412905.2005.9699008

[B207] Tan C. S. Isa N. M. Ismail I. Zainal Z. (2019). Agarwood induction: current developments and future Perspectives. Front. Plant Sci. 10, 122–135. 10.3389/fpls.2019.00122 30792732 PMC6374618

[B208] Tang X. Yuan Y. Zhang J. (2020). How climate change will alter the distribution of suitable Dendrobium habitats. Front. Ecol. Evol. 8, 536339–536356. 10.3389/fevo.2020.536339

[B209] Taura L. Gudžinskas Z. (2024). What factors determine the natural fruit Set of *Cephalanthera longifolia* and *Cephalanthera rubra*? Diversity 16 (6), 333–357. 10.3390/d16060333

[B210] Terletskaya N. V. Turzhanova A. S. Khapilina O. N. Zhumagul M. Z. Meduntseva N. D. Kudrina N. O. (2023). Genetic diversity in natural populations of *Rhodiola* species of different adaptation strategies. Genes (Basel) 14 (4), 794–808. 10.3390/genes14040794 37107552 PMC10137911

[B211] The World Health Organization (WHO) (1993). Guidelines on the conservation of medicinal plants. Gland, Switzerland: WWF – World Wide Fund for Nature. IUCN -The World Conservation Union; WWF-World Wide Fund for Nature: The International Union for Conservation of Nature and Natural Resources (IUCN), Gland, Switzerland, in partnership with The World Health Organization (WHO). Geneva, Switzerland.

[B212] Thompson I. Lim T. Turjaman M. (2022). Expensive, Exploited, and Endangered. A review of the agarwood-producing genera Aquilaria and Gyrinops. CITES considerations, trade patterns, conservation, and management ITTO Technical. Available at: https://cites.org/sites/default/files/documents/E-CoP19-Inf-12.pdf.

[B213] Thulin M. Decarlo A. Johnson S. P. (2019). *Boswellia occulta* (Burseraceae), a new species of frankincense tree from Somalia (Somaliland). Phytotaxa 394, 219–226. 10.11646/phytotaxa.394.3.3

[B214] UN Environmental Programme (2024). Landmark UN report: the world’s migratory species of animals are in decline, and the global extinction risk is increasing. Available at: https://www.unep.org/news-and-stories/press-release/landmark-un-report-worlds-migratory-species-animals-are-decline.

[B215] USDA. China (2022). American Ginseng approved for use as a food ingredient under Guangdong pilot program. Washington, DC: USDA Foreign Agricultural Service. Available at: https://fas.usda.gov/data/china-american-ginseng-approved-use-food-ingredient-under-guangdong-pilot-program.

[B216] USFWS (2005). Convention permit applications for wild American ginseng harvested in 2005. Washington, D.C.: U.S. Fish and Wildlife Service USFWS. Available at: https://www.fws.gov/international-affairs/permits/ginseng. 20240

[B217] Van den Bosch K. Witkowski E. T. F. Thompson D. I. Cron G. V. (2023). Reproductive ecology offers some answers to the pepperbark tree persistence puzzle in the Kruger National Park, South Africa. Glob. Ecol. Conservation 41, e02330. 10.1016/j.gecco.2022.e02330

[B218] Van der Voort M. E. (2005). An ecological study of Panax quinquefolius in central Appalachia: seedling growth, harvest impacts and geographic variation in demography. Morgantown, West Virginia: West Virginia University, 167.

[B219] Van Wyk B. E. (2011). The potential of South African plants in the development of new medicinal products. South Afr. J. Bot. 77 (4), 812–829. 10.1016/j.sajb.2011.08.011

[B220] Van Wyk B.-E. Gericke N. (2000). People's plants: a Guide to useful plants of southern Africa. Pretoria: Briza Publications, 351.

[B221] Van Wyk B.-E. Van Oudtshoorn B. Gericke N. (2009). Medicinal plants of South Africa. Revised and expanded edition. Pretoria: Briza Publications, 336.

[B222] Veeman T. S. Cunningham A. B. Kozanayo W. (2014). “The economics of production of rare medicinal species introduced in southwestern Zimbabwe: Warburgia salutaris,” in Bark Use, management and commerce in Africa. Editor Cunningham A. B. (New York: The New York Botanical Garden Press), 179–188.

[B223] Wang C. Peng D. Liu Y. Wu Y. Guo P. Wei J. (2021). Agarwood Alcohol extract protects against Gastric ulcer by Inhibiting Oxidation and inflammation. Evid. Based Complementary Altern. Med. 2021, 9944685–9944696. 10.1155/2021/9944685 PMC8464430 34580595

[B224] Wang Z. Wang T. Hu J. Jiao H. Jin Y. Sun J. (2024b). Comparisons of wild and cultivated American ginseng (*Panax quinquefolius* L.) genomes provide insights into changes in root growth and metabolism during domestication. Plant Biotechnolology J. 22 (7), 1963–1965. 10.1111/pbi.14316 PMC11182593 38446695

[B225] Wang Z. Wang T. Zhang X. Wang J. Yang Y. Sun Y. (2024a). Biodiversity conservation in the context of climate change: facing challenges and management strategies. Sci. Total Environ. 937, 173377–173391. 10.1016/j.scitotenv.2024.173377 38796025

[B226] Wani T. A. Kaloo Z. A. Dangroo N. A. (2022). Aconitum heterophyllum Wall. ex Royle: a critically endangered medicinal herb with rich potential for use in medicine. J. Integr. Med. 20 (2), 104–113. 10.1016/j.joim.2021.12.004 34996731

[B227] Wen J. Zhou L. Liu L. He Y. (2022). Analysis of the impact of climate change on the distribution and active compound content of the plateau medicinal plant *Nardostachys jatamansi* (D. Don) DC. Industrial Crops Prod. 187, 115438–115447. 10.1016/j.indcrop.2022.115438

[B228] White D. (2000). Raiding Mother Earth's treasure chest. Lady-Slipper Newsl. Ky. Native Plant Soc. 15 (2-3-Summer), 4.

[B229] Williams V. L. Victor J. E. Crouch N. R. (2013). Red Listed medicinal plants of South Africa: status, trends, and assessment challenges. South Afr. J. Bot. 86, 23–35. 10.1016/j.sajb.2013.01.006

[B230] Williams V. L. Witkowski E. T. F. Balkwill K. (2007). Relationship between bark thickness and diameter at breast height for six tree species used medicinally in South Africa. South Afr. J. Bot. 73 (3), 449–465. 10.1016/j.sajb.2007.04.001

[B231] Xiao M. (2000). The Canadian ginseng industry: preparing for the 21st century. Ottawa, Canada: The Canadian Ginseng Industry, 7. Agrifood Canada -The Canadian Ginseng Industry - Preparing for the 21st Century - 1999.h11/8/01.

[B232] Xu Y. Huang J. Lu X. Ding Y. Zang R. (2019). Priorities and conservation gaps across three biodiversity dimensions of rare and endangered plant species in China. Biol. Conserv. 229, 30–37. 10.1016/j.biocon.2018.11.010

[B233] Yang M. Sun L. Yu Y. Zhang H. Malik I. Wistuba M. (2023). Predicting the potential geographical distribution of *Rhodiola* L. In China under climate change Scenarios. Plants (Basel) 12 (21), 3735–3749. 10.3390/plants12213735 37960089 PMC10648157

[B234] Yesuf G. U. Brown K. A. Walford N. S. Rakotoarisoa S. E. Rufino M. C. (2021). Predicting range shifts for critically endangered plants: is habitat connectivity irrelevant or necessary? Biol. Conserv. 256, 109033–109046. 10.1016/j.biocon.2021.109033

[B235] You J. Qin X. Ranjitkar S. Lougheed S. C. Wang M. Zhou W. (2018a). Response to climate change of montane herbaceous plants in the genus *Rhodiola* predicted by ecological niche modelling. Sci. Rep. 8 (1), 5879–5891. 10.1038/s41598-018-24360-9 29651147 PMC5897335

[B236] You J. Qin X. Ranjitkar S. Lougheed S. C. Wang M. Zhou W. (2018b). Response to climate change of montane herbaceous plants in the genus *Rhodiola* predicted by ecological niche modelling. Sci. Rep. 8 (1), 5879–5891. 10.1038/s41598-018-24360-9 29651147 PMC5897335

[B237] Young B. E. Dubois N. S. Rowland E. L. (2015). Using the climate change vulnerability index to inform adaptation planning: lessons, innovations, and next steps. Wildl. Soc. Bull. 39 (1), 174–181. 10.1002/wsb.478

[B238] Yu C. Wang C. Z. Zhou C. J. Wang B. Han L. Zhang C. F. (2014). Adulteration and cultivation region identification of American ginseng using HPLC coupled with multivariate analysis. J. Pharm. Biomed. Analysis 99, 8–15. 10.1016/j.jpba.2014.06.031 PMC4151300 25044150

[B239] Zhang J. Lu J. Zhu Y. Huang Q. Qin L. Zhu B. (2022). Rhizosphere microorganisms of *Crocus sativus* as antagonists against pathogenic *Fusarium oxysporum* . Front. Plant Sci. 13, 1045147–1045160. 10.3389/fpls.2022.1045147 36483959 PMC9722746

[B240] Zou H. Chen B. Zhang B. Zhou X. Zhang X. Wang J. (2023). Conservation planning for the endemic and endangered medicinal plants under the climate change and human disturbance: a case study of *Gentiana manshurica* in China. Front. Plant Sci. 14, 1184556–1184576. 10.3389/fpls.2023.1184556 37564387 PMC10410459

